# A Randomized Controlled Trial on the Effects of a 12-Week High- vs. Low-Intensity Exercise Intervention on Hippocampal Structure and Function in Healthy, Young Adults

**DOI:** 10.3389/fpsyt.2021.780095

**Published:** 2022-01-21

**Authors:** Antonia Kaiser, Liesbeth Reneman, Michelle M. Solleveld, Bram F. Coolen, Erik J. A. Scherder, Linda Knutsson, Atle Bjørnerud, Matthias J. P. van Osch, Jannie P. Wijnen, Paul J. Lucassen, Anouk Schrantee

**Affiliations:** ^1^Department of Radiology and Nuclear Medicine, Amsterdam University Medical Center, University of Amsterdam, Amsterdam, Netherlands; ^2^Department of Biomedical Engineering and Physics, Amsterdam University Medical Centers, University of Amsterdam, Amsterdam, Netherlands; ^3^Department of Clinical Neuropsychology, Vrije Universiteit Amsterdam, Amsterdam, Netherlands; ^4^Russell H. Morgan Department of Radiology and Radiological Science, Johns Hopkins University School of Medicine, Baltimore, MD, United States; ^5^Department of Medical Radiation Physics, Lund University, Lund, Sweden; ^6^F.M. Kirby Research Center for Functional Brain Imaging, Kennedy Krieger Institute, Baltimore, MD, United States; ^7^Department of Diagnostic Physics, Oslo University Hospital, Oslo, Norway; ^8^Department of Physics, University of Oslo, Oslo, Norway; ^9^Department of Radiology, Leiden University Medical Center, Leiden, Netherlands; ^10^Department of Radiology, University Medical Center Utrecht, Utrecht, Netherlands; ^11^Swammerdam Institute for Life Sciences, University of Amsterdam, Amsterdam, Netherlands; ^12^Center for Urban Mental Health, University of Amsterdam, Amsterdam, Netherlands

**Keywords:** hippocampus, exercise, MRI, multimodal, vasculature, perfusion, neuro-metabolites, angiogenesis

## Abstract

Physical exercise affects hippocampal structure and function, but the underlying neural mechanisms and the effects of exercise intensity remain incompletely understood. Therefore, we undertook a comprehensive, multi-modal 3T and 7T MRI randomized controlled trial (Netherlands Trial Register - NL5847) in which we randomized 52 young, non-athletic volunteers to a 12-week low- or high-intensity exercise program. Using state-of-the-art methods, we investigated changes in hippocampal volume, as well as changes in vasculature, neuro-metabolites, and peripheral growth factors as potential underpinnings. Cardiorespiratory fitness improved over time (*p* < 0.001), but no interaction with exercise intensity was found (*p* = 0.48). Accordingly, we did not observe significant interactions between exercise condition and time on MRI measures (all *p* > 0.06). However, we found a significant decrease in right hippocampal volume (*p* < 0.01), an increase in left hippocampal glutathione (*p* < 0.01), and a decrease of left hippocampal cerebral blood volume (*p* = 0.01) over time, regardless of exercise condition. Additional exploratory analyses showed that changes in brain-derived neurotrophic factor (*p* = 0.01), insulin-like growth-factor (*p* = 0.03), and dorsal anterior cingulate cortex N-acetyl-aspartate levels (*p* = 0.01) were positively associated with cardiorespiratory fitness changes. Furthermore, a trend toward a positive association of fitness and gray-matter cerebral blood flow (*p* = 0.06) was found. Our results do not provide evidence for differential effects between high-intensity (aerobic) and low-intensity (toning) exercise on hippocampal structure and function in young adults. However, we show small but significant effects of exercise on hippocampal volume, neurometabolism and vasculature across exercise conditions. Moreover, our exploratory results suggest that exercise might not specifically only benefit hippocampal structure and function, but rather has a more widespread effect. These findings suggest that, in agreement with previous MRI studies demonstrating moderate to strong effects in elderly and diseased populations, but none to only mild effects in young healthy cohorts, the benefits of exercise on the studied brain measures may be age-dependent and restorative rather than stimulatory. Our study highlights the importance of a multi-modal, whole-brain approach to assess macroscopic and microscopic changes underlying exercise-induced brain changes, to better understand the role of exercise as a potential non-pharmacological intervention.

## Introduction

Physical exercise can have numerous positive effects on our body and brain; including reductions in the risk for cardiovascular disease, stroke, and obesity. Furthermore, it has been found to promote brain plasticity and positively affect brain structure and function in both rodents and humans (1–6). Therefore, the possibility to use physical activity to improve brain health has received much attention lately as a low-cost and easy to apply, non-pharmacological intervention (7, 8). So far, however, the exact underlying mechanisms by which exercise can benefit the brain, and what role exercise intensity plays, have remained incompletely understood.

The first studies investigating brain correlates of exercise-induced changes sought to determine structural brain alterations. Using magnetic resonance imaging (MRI), multiple cross-sectional and prospective-longitudinal studies in humans have shown that high-intensity aerobic exercise increased or normalized age-related decreases in brain volume, particularly in the hippocampus (9, 10). In their meta-analysis, Firth et al. found the most substantial exercise effects in older adults (11), even though some studies also reported rapid hippocampal volume increases in younger adults (12).

Volume changes alone lack information on biological substrates of exercise-related changes (13). Both animal and human studies have proposed several underlying mechanisms (14, 15), such as changes in perfusion, as measured with cerebral blood flow (CBF), vascularization as measured with cerebral blood volume (CBV), synaptic plasticity and neurogenesis as estimated by neuro-metabolite concentrations, and other molecular and cellular changes (3, 16–21). For instance, rodent studies have shown exercise-induced increases in angiogenesis and neurogenesis (13, 22–24). Physical exercise was further shown to alter specific neuro-metabolites; Biedermann et al. and Wagner et al. reported decreased right hippocampal glutamate (Glu) levels of mice and humans after prolonged exercise (25, 26), and similar results were found in the human occipital cortex (27) and the rat striatum (28). Cross-sectional studies have further associated higher fitness of endurance-trained, middle-aged adults with higher N-acetyl aspartate (NAA) levels in their frontal cortex (29, 30).

In summary, physical exercise in both animals and humans influences various mechanisms that may alter brain structure (6, 31). In this respect, Thomas et al. were one of the first to investigate volume changes in young, healthy adults in a multi-modal approach (12). They used several neuroimaging measures of volume, vasculature, and microstructure, and specifically found a temporary increase in volume and myelination, but no vascular changes. So far, the role of exercise intensity has received little attention (15, 32), even though a recent meta-analysis stressed the importance of high-intensity training [heart-rate (HR) > 80% of maximum HR] for improving fitness in younger adults (33, 34).

Therefore, we here undertook a comprehensive, multi-modal study to compare the effects of a 12-week high- vs. low-intensity exercise paradigm in young, healthy, but otherwise non-athletic volunteers. We studied exercise-induced changes in hippocampal volume, and additionally, its potential underpinnings, like changes in angiogenesis, synaptic plasticity, neurogenesis, and peripheral growth factors. Because earlier studies had indicated that certain changes only occur in specific hippocampal subfields (35), we further explored changes in hippocampal subfield volume and relations of all measures to individual changes in cardiorespiratory fitness. We used 3T MRI to study outcomes related to vascular changes and 7T MRI to obtain high-resolution anatomical delineation of hippocampal subfields and reliable quantification of various neuro-metabolites (36). To study exercise intensity and control for baseline differences in fitness, we randomized participants, after stratification for age, sex, and baseline VO_2_max, to a high-intensity, aerobic exercise condition, or a low-intensity, stretching and toning (active control) exercise condition.

Based on earlier literature, we hypothesized that cardiorespiratory fitness and hippocampal volume increases would occur in the high-, but not low-intensity exercise condition. Based on an increase in hippocampal volume, we furthermore expected changes in vascularization, as measured with cerebral blood flow (CBF), and cerebral blood volume (CBV), along with vascular endothelial growth factor (VEGF) concentrations, as a marker for vascular maintenance and remodeling. Moreover, neuronal remodeling was expected, estimated through changes in neuro-metabolite concentrations of NAA, glutathione (GSH), glutamate (Glu), and glutamine (Gln), and brain-derived neurotrophic factor (BDNF) as well as peripheral insulin-like growth factor-1 (IGF-1), as markers for neuronal development. We chose the dorsal anterior cingulate cortex (dACC) as a control region and regarded whole-brain gray matter (GM) changes as evidence for non-specific effects.

## Materials and Methods

### Participants and Experimental Design

**Figure 1:**
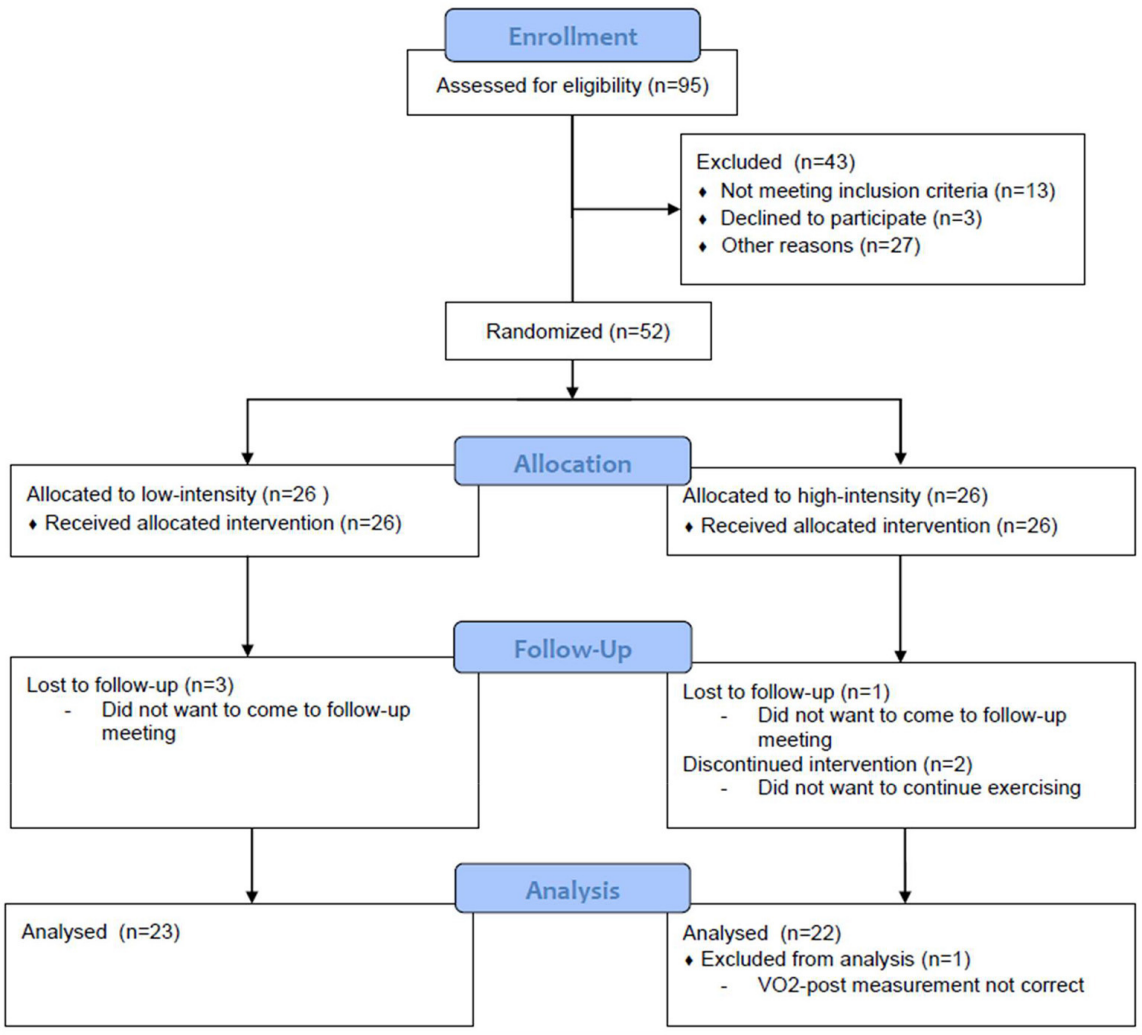
CONSORT flow chart.

**Figure 2:**
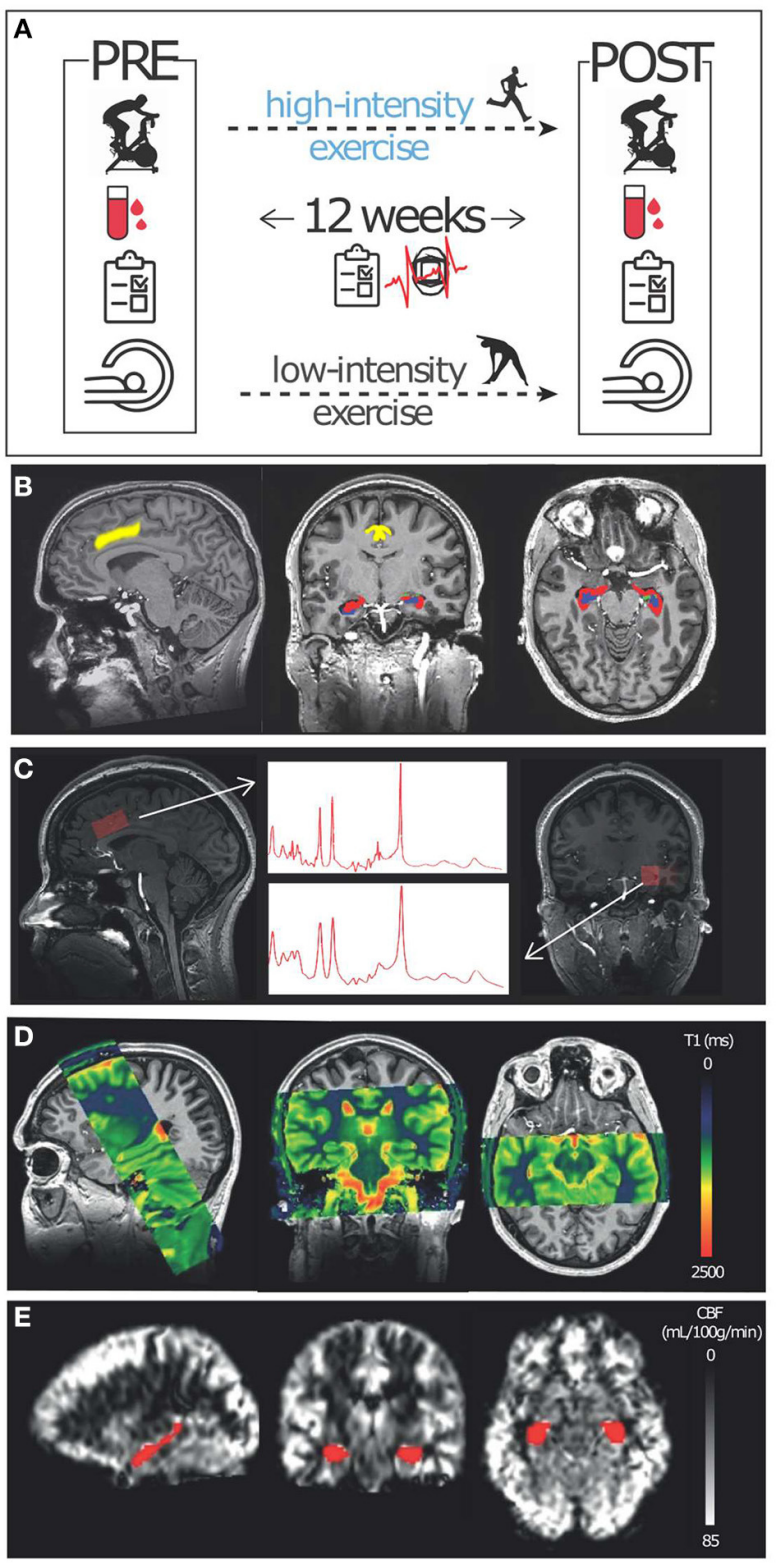
Study methods: **(A)** Participants were enrolled in a 12-week low- (active control) and high-intensity exercise intervention. Several measures, including a cardiorespiratory fitness test (VO_2_max), and peripheral growth factors (blood sampling) were conducted before (PRE) and after (POST) the exercise intervention. Additionally, HR, exercise frequency, and exercise questionnaires were collected during the intervention. Furthermore, several MRI measures were collected before and after the exercise regime: **(B)** T1- and T2-weighted scans were conducted at 7T for segmentation purposes. **(C)** Single voxel spectroscopy was conducted at 7T in the dACC (left) and left hippocampus (right). **(D)** T1-mapping using a steady-state contrast-enhanced method was conducted at 3T to derive CBV and R1. **(E)** A pCASL sequence was used at 3T to obtain CBF values.

Participants were recruited through posters and online advertisements. We included 52 healthy, non-athletic volunteers (30 women and 22 men, aged 18–30 years old, Table 1) in a 3-month randomized, controlled trial (Netherlands Trial Register - NL5847; Figure 1). After stratification for age, sex, and baseline cardiorespiratory fitness, participants were randomized to a 12-week intervention of high- or low-intensity exercise training. Before and after the intervention, participants performed a maximal exercise test to measure cardiorespiratory fitness and underwent MRI measurements (Figure 2A).

**Table 1:** Participant characteristics and fitness measures.

	**High-intensity**		**Low-intensity**		Statistics*^2^	
	**Female (*N* = 11) Mean ±SD**	**Male (*N* = 11) Mean ±SD**	**Female (*N* = 13) Mean ±SD**	**Male (*N* = 10) Mean ±SD**	**Female**	**Male**
**Participant characteristics**						
Age (y)	23.87 ± 2.59	22.09 ± 2.09	24 ± 2.58	24.64 ± 4.15	*t*_(23.92)_ = −0.14, *p* = 0.89	*t*_(14.88)_ = 1.81, *p* = 0.09
Education	7.00 ± 1.11	6.70 ± 1.49	7.31 ± 1.03	6.36 ± 2.01	*t*_(23.00)_ = −0.75, *p* = 0.46	*t*_(18.31)_ = 0.44, *p* = 0.67
Body mass index (kg/m^2^)	23.12 ± 3.19	23.55 ± 3.17	23.67 ± 2.51	23.46 ± 2.23	*t*_(23.70)_ = 0.52, *p* = 0.60	*t*_(17.95)_ = −0.08, *p* = 0.94
IQ estimate (DART)	106.47 ± 5.40	107.60 ± 6.33	107.21 ± 7.20	105.55 ± 7.41	*t*_(23.06)_ = −0.32, *p* = 0.76	*t*_(18.94)_ = 0.69, *p* = 0.50
**Fitness measures**						
VO_2_max pre (kg/mL/min)	33.71 ± 5.18	41.98 ± 7.44	34.44 ± 4.75	41.78 ± 6.22	*t*_(27.80)_ = −0.18, *p* = 0.85	*t*_(19.39)_ = −0.07, *p* = 0.95
VO_2_max post (kg/mL/min)	38.79 ± 5.25	45.25 ± 6.83	36.15 ± 6.14	43.59 ± 6.93	*t*_(23.44)_ = −1.18, *p* = 0.25	*t*_(18)_ = −0.54, *p* = 0.60
Max. heart rate pre (beats/min)	182.92 ± 8.36	192.12 ± 4.85	190.64 ± 7.97	187.03 ± 6.68	*t*_(27.94)_ = 2.59, *p* = 0.01	*t*_(18.18)_ = −2.01, *p* = 0.06
Max. heart rate post (beats/min)	185.08 ± 9.57	187.11 ± 6.68	190.10 ± 6.88	184.07 ± 6.68	*t*_(21.79)_ = 1.54, *p* = 0.14	*t*_(18.81)_ = −1.04, *p* = 0.31
Resistance pre (watts)	200.33 ± 33.03	294.09 ± 49.84	221.00 ± 31.58	276.82 ± 42.56	*t*_(27.94)_ = 1.75, *p* = 0.09	*t*_(19.52)_ = −0.87, *p* = 0.39
Resistance post (watts)	225.38 ± 30.38	330.45 ± 53.03	239.62 ± 29.04	291.50 ± 47.26	*t*_(23.95)_ = 1.22, *p* = 0.23	*t*_(18.99)_ = −1.78, *p* = 0.09
**Intervention**						
Duration of exercise (h)	32.44 ± 14.81	26.46 ± 7.13	31.69 ± 9.51	26.78 ± 14.69	*t*_(20.63)_ = −0.15, *p* = 0.88	*t*_(17.97)_ = −0.60, *p* = 0.56
Percent of hours with HR > 80% of max. HR^*1^	35.92 ± 17.11	35.84 ± 19.92	13.51 ± 9.39	4.53 ± 3.69	*t*_(18.04)_ = −4.21, *p* < 0.01	*t*_(10.83)_ = −5.11, *p* < 0.01

pre = pre-exercise intervention; post = post-exercise intervention; Education [Dutch System; (37)]: 0 = no education, 1 = Elementary School, 2 = VMBO, 3 = VMBO-T, 4 = MBO, 5 = HAVO, 6 = VWO, 7 = HBO, 8 = WO (University); Body Mass Index = weight (kg)/[height (m)]^2^; NLV = Dutch reading test estimating intelligence quotient (IQ); max. heart rate = max. heart rate measured during VO_2_max test; Resistance = max. resistance of ergometer during VO_2_max test; Duration of exercise = Hours spent exercising over the 12-week exercise intervention; *1 = Percentage of hours spent exercising with a HR above 80% of individuals max. HR (220-age); *2 = Independent samples t-tests, in case of non-normal distribution, data were transformed.

Participants that were classified as athletic based on the definition by Haddad Herdy and Uhlendorf (38), i.e., VO_2_max_males_ > 55 ml/kg/min, VO_2_max_females_ > 45 ml/kg/min, were excluded. Furthermore, participants who engaged in intensive sports (>3 times/week) were also excluded. In addition, we excluded participants based on the following criteria: BMI >30 kg/m^2^ (>class 1 obesity), MRI contra-indications, a history of chronic renal insufficiency, allergy to gadolinium-containing compounds, a history of psychiatric disorders, excessive smoking (>1 pack/day), excessive alcohol consumption (>21 units/week), or other regular drug use. Additionally, females were only included if they were on hormonal contraceptives to control for the effects of the hormonal cycle. We obtained written informed consent from all participants, and the study was approved by the local Medical-Ethical Committee of the Amsterdam University Medical Centre, University of Amsterdam (NL55943.018.15).

### Exercise-Intervention

All participants were enrolled in an exercise program for 12 weeks, in which they were instructed to exercise three times a week for 45 min (1, 39) at the university sports center (USC). Their presence and active engagement were monitored by tracking their sports center visits using an automated fingerprint entrance system and by using weekly questionnaires on exercise duration and activities. Additionally, participants received a HR monitor (Polar, Finland) to measure HR during each training session (Table 1). Participants randomized to high-intensity exercise performed high-intensity interval training, targeting HR zones above 80% of their maximum HR. Participants randomized to low-intensity exercise performed stretching and toning exercises (active control condition), targeting HR zones under 60% of their maximum HR. In collaboration with the sports scientists of the USC, we provided a list of generic exercise group classes offered at the USC which were supervised by an experienced fitness instructor, that participants were allowed to choose from [Supplementary Material 1.5; Supplementary CERT Checklist (40)]. Participants that were engaged in physical activity before the study were instructed to do the recommended sports classes on top of their usual activities. For motivation purposes, participants were contacted regularly to check in on their progress and one experimenter joined them at least once during the intervention period to train with them (more detail in Supplementary Material 1.5). The Dutch version of the International Physical Activity Questionnaire [IPAQ; (41)] was used to measure physical activity during walking, intermediate and vigorous intensities before and after the intervention.

### Cardiorespiratory Fitness

Participants underwent a cardiopulmonary exercise test on an ergometer before and after the exercise intervention to assess individual cardiorespiratory fitness. After 2 min of rest (baseline measurement), an incremental bicycle protocol (which was dependent on weight and sex) was started with a 3-min warm-up period, followed by an increase in resistance (watts) every minute until maximal effort (maximum resistance) or exhaustion, which was immediately followed by a 2-min recovery period at 50 watts resistance. Breath-by-breath gas exchange measurement data were obtained to determine maximum oxygen uptake [VO_2_max (mL/kg/min)] (39). VO_2_max data were time-averaged using 10s intervals (26). For exercise tests to be considered maximal, participants had to reach both a plateau in VO_2_max and a respiratory exchange ratio of > 1.1 CO_2_/O_2_. VO_2_max tests took place at least 24 h before the MRI scans.

### MRI Acquisition

Participants were scanned on a 7T whole-body MR system (Philips, Best, The Netherlands) using a dual-channel transmit coil and a 32-channel receive head-coil, and on a 3T whole-body MR system (Philips, Best, The Netherlands) using a body transmit coil and a 32-channel receive head-coil. A 24h gap between the last workout and MRI scanning was ensured to minimize the potential influences of dehydration on brain volume and acute exercise effects (42).

#### 7T MRI

Whole-brain T1-weighted data were obtained with a sagittal 3D magnetization-prepared rapid gradient echo (MP-RAGE) sequence (TR/TE = 4.1/1.8 ms; TI = 1,300 ms; 0.9 × 0.9 × 0.9 mm3 isotropic voxels; flip-angle = 7°). T2-weighted data covering the hippocampus were obtained using a coronal multi-slice turbo spin-echo (TSE) sequence (TR/TE = 6,000/80 ms; voxel-size = 0.4 × 0.4 × 2 mm; flip-angle = 110°) (Figure 2B). Single voxel 1H-MRS data were collected from the left hippocampus and dACC with a semi-localized adiabatic selective refocusing (sLASER) sequence [TR/TE = 5,000/36 ms; FOCI pulses (43)]; to ensure correct adiabatic behavior of the FOCI pulses: B1 > 17 μT; bandwidth = 4 kHz; 2,048 data points; voxel-size = 30 × 15 × 15 mm; NSA_dACC_ = 64; NSA_hippocampus_ = 128 (Figure 2C; Supplementary Figure 1). Non-water suppressed spectra were obtained for quantification and eddy-current correction.

#### 3T MRI

To obtain CBV and myelination [R1 = (1/T1)], quantitative T1 measurements of the hippocampus and sagittal sinus blood were performed before and after gadolinium contrast administration [0.1 mL/kg, 1–2 mL/s followed by 20 mL saline (0.9% NaCl)] (Gadovist, Bayer B.V., Mijdrecht, The Netherlands). For brain T1-mapping, a 3D Look-Locker sequence with 40 inversion times was performed as described by Lindgren et al. (44), in a coronal slab covering the hippocampus (Figure 2D) with the following parameters: TR/TE = 10/4 ms; flip-angle = 5°; TI = 110 ms, inter-shot TR = 6 s, resolution = 1.15 × 1.15 × 2 mm, acquisition time = 6 min. Blood T1 values were obtained using a single-slice multi-time-point inversion recovery sequence planned perpendicular to the posterior sagittal sinus with parameters: TR/TE = 110/16 ms, flip angle = 95°; resolution = 1.5 × 1.5 mm; slice-thickness = 2 mm. To obtain whole-brain CBF measures we used a gradient-echo single-shot EPI pseudo-continuous arterial spin labeling (pCASL) sequence with background suppression (TR/TE = 4,091/16 ms; label-duration = 1,650 ms, post-label delay = 1,525 ms; voxel-size = 3 × 3 × 5 mm) (Figure 2E). For CBF quantification, an additional M0 scan was acquired using the same imaging parameters, except for the TR = 2,000 ms, and without labeling and background suppression.

### MRI Data Analysis

#### Volume

Using both T1-weighted and T2-weighted scans, segmentations of the hippocampus were performed in native space using Automatic Segmentation of Hippocampal Subfields (ASHS) software (45, 46). This method automatically generates segmentations based on a segmentation atlas (45) with a machine-learning algorithm using similarity-weighted voting and learning-based bias-correction techniques (Figure 2B). The following subfields were defined: whole hippocampus, consisting of CA1, CA2, CA3, CA4, DG, subiculum, head, tail, entorhinal cortex, and cysts. Segmentation of the dACC was performed with Freesurfer v.5.3.0 (47). Gray matter (GM) and white matter (WM) segmentations were performed with SPM12.

For the main analyses, all measures were calculated for the whole left and right hippocampus. The dACC was used as a control region, and whole-brain GM changes were regarded as region-unspecific effects. Further exploratory analyses involved volume measures of hippocampal subfields: CA1, CA3, and dentate gyrus, and hippocampal GM and WM.

#### 1H-MRS

Pre-processing included optimized coil combination, eddy current correction, and spectral registration (48). Spectra were fitted using LCModel with a simulated basis set with a measured macromolecular baseline (Supplementary Methods 1.1). Metabolite concentrations for glutamate, glutamine, glutathione (GSH), and N-acetyl-aspartate (NAA) were calculated using water-scaling and were corrected for partial volume effects using the tissue volume fractions (49). T1-weighted scans were segmented using SPM12 to determine the contributions of GM, WM, and CSF to each voxel. Spectral quality measures calculated with LCModel, and signal-to-noise ratio (SNR > 30), linewidth (FWHM > 19 Hz), and Cramér–Rao lower bounds (CRLB ≤ 40), were used to exclude lower-quality spectra (50, 51).

#### CBF

ASL post-processing was performed using ExploreASL (52). Motion was estimated, spike frames > mean + 3 standard deviations (SD) were deleted, and motion estimation was repeated. ASL perfusion-weighted images were registered to GM-tissue probability maps of each participant using six-degrees-of-freedom (DOF). Label and control images were pairwise subtracted (M), corrected for slice gradients, and averaged. CBF was calculated using the single-compartment model (53), using a separate M0 image and individual hematocrit values that were derived from blood samples to calculate T1-blood values. Before and after quantification, voxel-based outlier rejection was applied. GM-tissue probability maps were normalized using Diffeomorphic Anatomical Registration analysis using Exponentiated Lie algebra (DARTEL), and T1-to-MNI transformation fields were applied to CBF maps (54). Median ROI CBF was based on voxels remaining after excluding voxels with CBF values exceeding 2.5 times the mean CBF over the entire volume, assumed to originate from large vessels. Thresholded left and right hippocampal masks (Harvard-Oxford Subcortical Structural Atlas) were masked for GM, and median ROI CBF values were calculated per participant.

#### CBV and Myelination

ROI averaged Look-Locker signal time curves were generated from different ROIs. T1 values for each ROI were calculated using a 3-parameter fit of the Look-locker signal equation (55). For determination of blood T1 values, 5 pixels that showed the highest average signal intensity, averaged over the last ten inversion times, within the sagittal sinus, were selected. Subsequently, T1 was determined by averaging signals from different combinations of pixels and choosing combinations that resulted in the lowest T1 fit-error based on a 3-parameter fit of the multi-timepoint inversion recovery curve. Finally, CBV was calculated using equations by Lindgren et al. with brain tissue density = 1.04 g/ml, hematocrit levels in large vessels = 0.45, and hematocrit levels in small vessels = 0.25, and GM CBV was corrected for the expected fast-water exchange-effects (44, 56, 57). Hippocampal values were consequently expressed relative to GM values. Furthermore, R1 values were obtained for left and right hippocampal WM by calculating 1/T1 to estimate myelination (58).

### Peripheral Neurotrophic Factors

Blood samples were collected before the MRI measurements, pre and post-intervention, to obtain: 1) brain-derived neurotrophic factor (BDNF) levels as a proxy for exercise effects on hippocampal neuronal health, plasticity, and possibly neurogenesis (59–61); 2) insulin-like growth factor 1 (IGF) as a proxy for cell proliferation and the inhibition of cell death (62), 3) free vascular endothelial growth factor (VEGF) levels, as a prime regulator of angiogenesis (63); and 4) hematocrit levels. For BDNF and IGF, a total of 4 mL serum was collected (15 min centrifugation at 1,000 × g). For VEGF, 8 mL serum was collected in PECT tubes (64) through an open system, drop by drop, without using a tourniquet (60 min centrifugation at 4°C at 1,700 × g). All samples were aliquoted and stored at −80°C. Growth factors were quantified using enzyme-linked immunosorbent assays (ELISAs) according to the manufacturer's instructions (R&D Systems; DVE00 for VEGF; DBD00 for BDNF; DG100 for IGF), and optical densities were converted into concentrations using an LP4 logistic fit (Graphpad Prism 5).

### Statistical Analysis

Sample size calculations can be found in the Supplementary Methods (1.4). All data were checked for normality and, in the case of non-normality, transformed accordingly. To account for missing data and the longitudinal nature of the trial, linear mixed-effects models were used to investigate the condition (high- vs. low-intensity exercise) × time (pre- vs. post-intervention) interaction effects in Rv.3.5.3 (65) using the lme4 package (66). Sex (female vs. male) was tested as a possible predictor but did not contribute to any of the models. Model selection was based on an adjusted top-down procedure, in which the resulting models were compared using the Bayesian information criterion (BIC), and subsequently, the model best capturing the data was reported using χ^2^ tests and BICs (67, 68). Bayes factors (BF) were calculated, indicating the strength of evidence, using BIC approximation (69). The evidence categories of Wetzels et al. were used (70) (Supplementary Table 1). We regarded changes in cardiovascular fitness and hippocampal and dACC volume as primary hypotheses, and changes in neuro-metabolites, CBV, myelination, CBF, and neurotrophic factors as secondary hypotheses.

Statistical tests regarding the interaction effects were corrected for multiple comparisons within modalities using Sidak's correction: α^*^ = 1 – (1 – α)^∧^(1/m), with α = 0.05 and m being the number of interaction and main effects (m = 3), which resulted in an α^*^ = 0.02. Additionally, Tukey corrected *t*-tests were used as *post-hoc* tests.

Furthermore, exploratory analyses testing associations of all variables with changes in VO_2_max were conducted using linear models in R, including the baseline measure of the explanatory variables and VO_2_max as covariates (α = 0.05). We additionally tested exercise-condition as a possible covariate, which did not contribute to the model. Mean and standard deviation per time-point per variable are reported in Supplementary Table 4.

## Results

Six participants dropped out during the exercise program and one participant was removed from the analysis because of incomplete VO_2max_ data. Therefore, the low-intensity condition consisted of 10 males and 13 females, the high-intensity condition of 11 males and 11 females. Conditions did not differ in age, sex, education, IQ-estimation, VO_2_max, or BMI at baseline (Table 1).

### Cardiorespiratory Fitness

**Figure 3:**
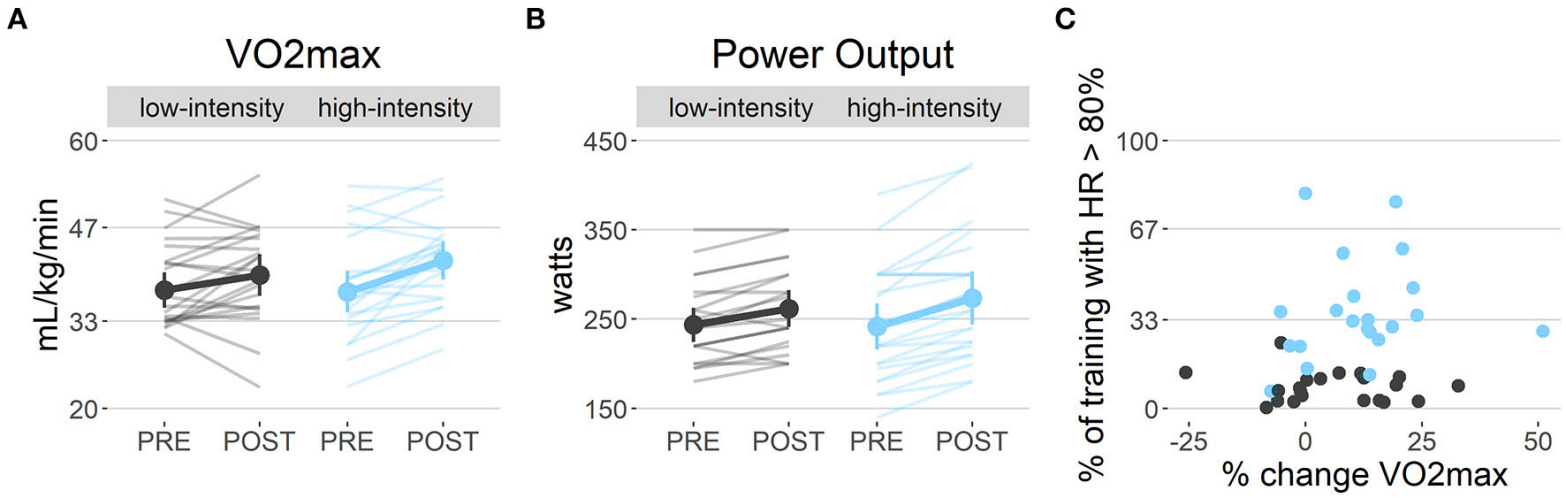
Cardiorespiratory fitness: **(A)** Cardiorespiratory fitness (VO_2_max) was found to increase over time irrespective of the exercise group (*p* < 0.01), even though *post-hoc* tests show only a significant increase in the high-intensity group (*p* < 0.01). **(B)** The ergometer power output during the VO_2_max test increased over time irrespective of the exercise group (*p* < 0.01), with the *post-hoc* test showing a significant increase in both groups (both *p* < 0.01). **(C)** Even though participants in the high-intensity group spent significantly more time in the target HR zone (80% of max. HR) than the low-intensity group (*p* < 0.01), the hours spent exercising was not associated with changes in fitness (*p* = 0.19).

Hours spent exercising demonstrated high compliance with the exercise program in both exercise groups (Table 1). As expected, participants in the high-intensity condition spent significantly more time exercising in the intended higher HR regime than the low-intensity condition, which did not explain the change in VO_2_max [*t*_(40)_ = 1.34, *p* = 0.19; Figure 3C]. The low- and high-intensity exercise groups did not show a significant change from pre- to post-intervention on the total score or scores for walking and intermediate intensity activities as measured with the IPAQ questionnaire. However, a significant condition x time effect was found on vigorous-intensity activities [χ^2^_(1)_ = 5.46, *p* = 0.02], indicating an increase in the high-intensity group but not in the low-intensity group (Supplementary Results 2.3). Nevertheless, contrary to our expectations, we found no condition x time effect on VO_2_max; instead, we found decisive evidence (BF > 100) for an effect of time [χ^2^_(1)_ = 15.43, *p* < 0.001; low-intensity: 4.7%, high-intensity: 12.65% change] (Figure 3A). Nevertheless, *post-hoc* tests revealed only a significant increase in the high-intensity condition [low: *t*_(49)_ = 1.72, *p* = 0.09; high: *t*_(49)_ = 4.20, *p* < 0.01]. In line with the results on VO_2_max, we found no interaction effect, but decisive evidence (BF > 100) for a main effect of time [χ^2^_(1)_ = 38.92, *p* < 0.001] on the maximal resistance attained. *Post-hoc* tests revealed a significant increase in both conditions [*t*_(23)_ = 4.67, *p* < 0.01; *t*_(24)_ = 7.02, *p* < 0.01; Figure 3B). No group effects on HR during the VO_2_max test were found (Supplementary Table 2).

### MRI

#### Volume

**Figure 4:**
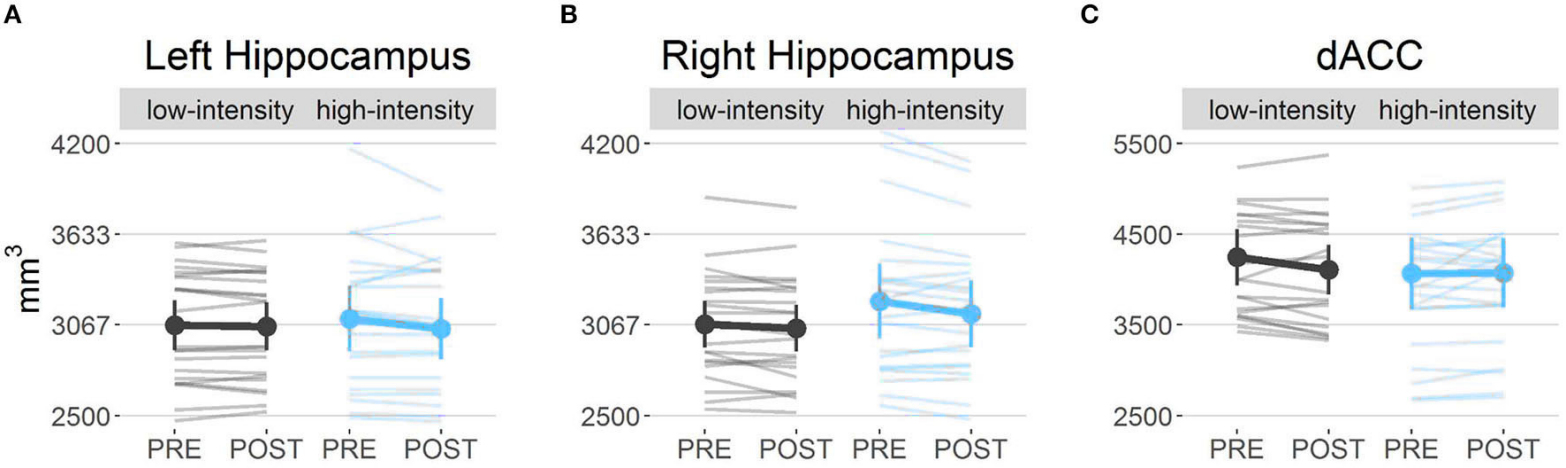
Volume measures: **(A)** Left hippocampal volume did not show any differences over time. **(B)** Right hippocampal volume decreased over time, irrespective of the exercise group (*p* < 0.01). *Post*-*hoc* tests revealed only a significant decrease in the high-intensity exercise group (*p* = 0.03). **(C)** The control region, the dorsal anterior cingulate cortex (dACC), did not show any significant changes over time.

For left and right hippocampal volume, one baseline scan had to be removed from the analysis due to incomplete hippocampal coverage of the T2-weighted scan. Left and right hippocampal volumes were analyzed separately, based on previous literature reporting lateralized effects of exercise (71, 72). We found no interactions between condition and time for either left and right whole hippocampal volume. However, we found substantial evidence (BF = 4.48) for a negative main effect of time in the right hippocampus [χ^2^_(1)_ = 7.51, *p* < 0.01]. *Post-hoc* tests further revealed only a significant decrease in the high-intensity condition [*t*_(47)_ = 2.22, *p* = 0.03; Figures 4A,B]. Consequently, we sought to determine whether this change was specific to a certain hippocampal subfield but found no significant effects (Supplementary Table 2). We did not find changes in volume in our control region, the dACC (Figure 4C).

#### 1H-MRS

**Figure 5:**
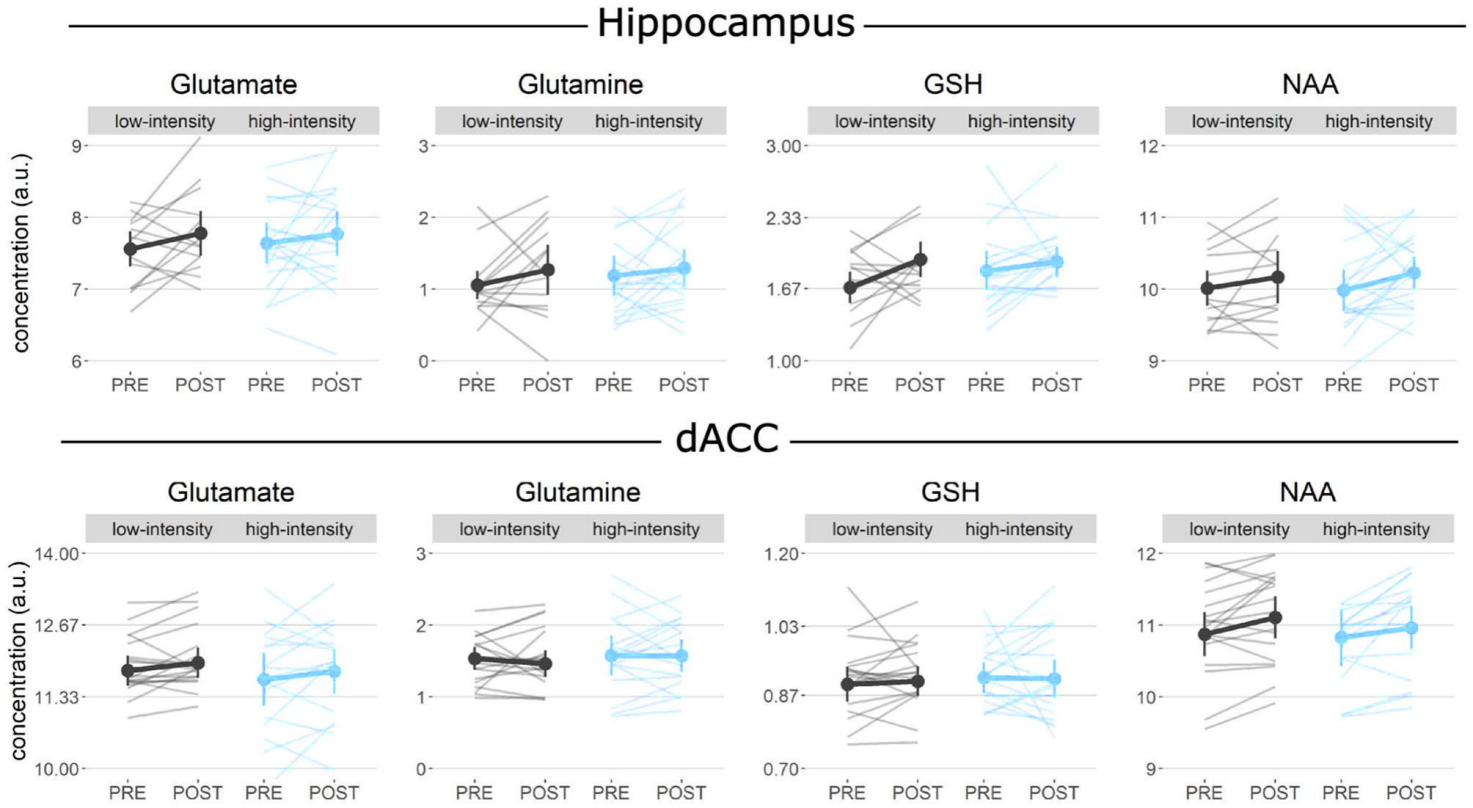
1H-MRS: GSH in the hippocampus was found to increase over time, irrespective of the exercise group (*p* < 0.01). No other metabolites in the hippocampus or the control region, dorsal anterior cingulate cortex (dACC), were found to change over time.

We were unable to obtain 1 baseline and 1 post spectrum in the left hippocampus and 7 baseline dACC spectra due to technical difficulties. Due to further exclusion based on stringent quality control measures (Supplementary Results 2.1) a total of 42 baseline and 43 post hippocampal spectra, and 40 baseline and 51 post dACC high-quality spectra were included in the analyses. No condition-by-time interactions were found for any neuro-metabolites investigated, i.e., glutamate, glutamine, glutathione, and NAA, in the hippocampus and the dACC (Supplementary Table 2). However, we found strong evidence (BF = 11.19) for increased GSH in the left hippocampus across conditions [main effect of time: χ^2^_(1)_ = 9.21, *p* < 0.01] (Figure 5).

#### Vascularization and Myelination

**Figure 6:**
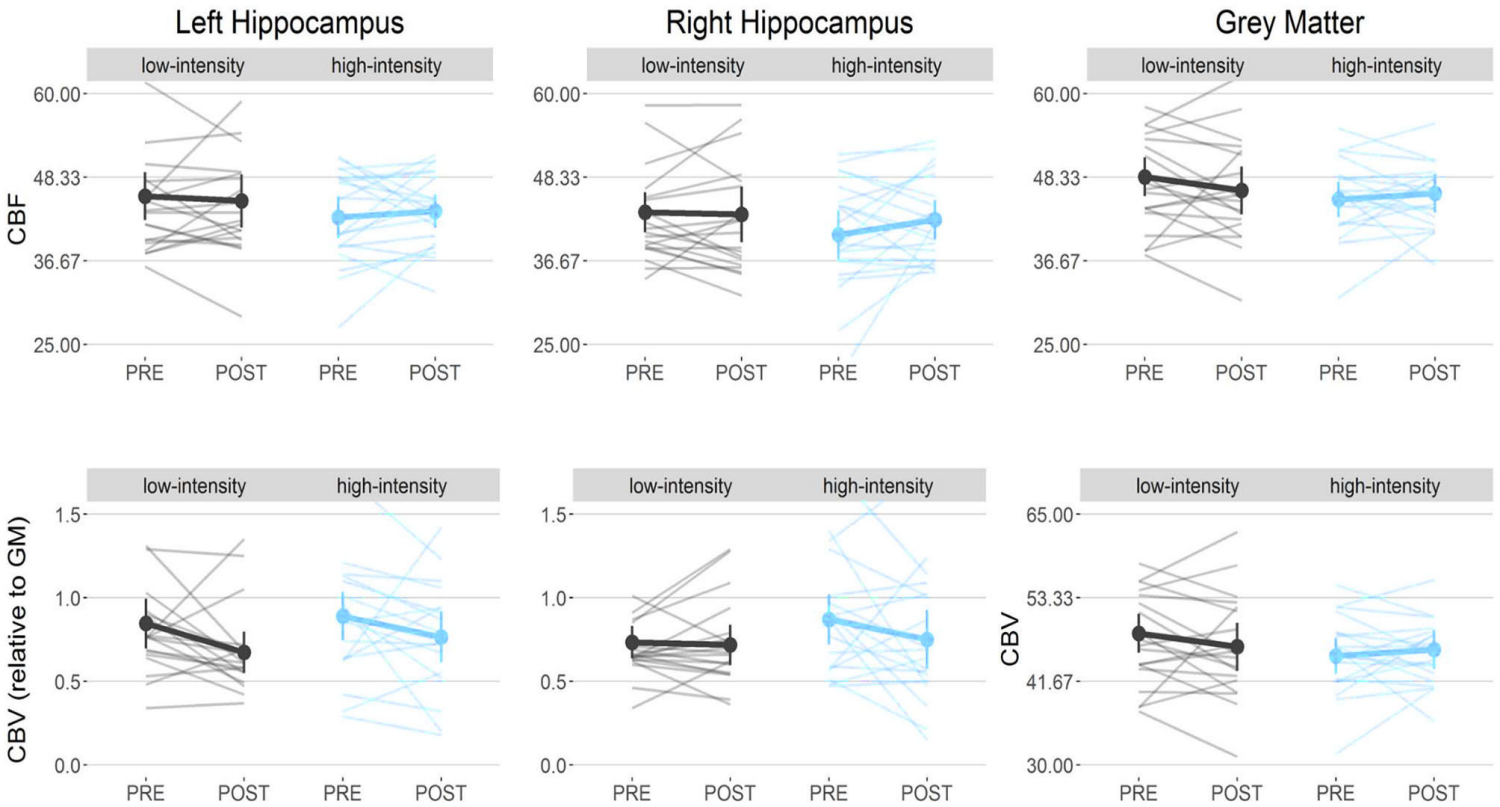
Vasculature: No vascular changes were found over time. CBV, Cerebral blood volume; CBF, Cerebral blood flow.

No condition-by-time interactions were found for CBF and CBV, in the left and right hippocampus, and GM (Supplementary Table 2; Figure 6). However, anecdotal evidence (BF = 2.79) for a decrease of CBV in the left hippocampus was found [main effect of time: χ^2^_(1)_ = 5.97, *p* = 0.01], with *post-hoc* tests showing a slight reduction in CBV in the low-intensity condition [*t*_(43)_ = 1.94, *p* = 0.05].

No condition-by-time interactions were found for R1 in the left and right hippocampal WM (Supplementary Table 2).

### Peripheral Neurotrophic Factors

We found no interaction effect of condition and time, and no main effects of time for BDNF, VEGF, or IGF levels (BF < 100^−1^) (Supplementary Table 2). No significant interaction effect of condition and time was found for hematocrit [χ^2^_(2)_ = 0.03, *p* = 0.98], but a main effect of time was found, indicating an increase in both exercise groups [χ^2^_(1)_ = 12.90, *p* < 0.01; BF = 8.4].

### Regression Analyses

#### MRI

Regression analyses demonstrated no association between changes in hippocampal or dACC volume and change in VO_2_max [left: *F*_(1, 41)_ = 0.17, *p* = 0.95; right: *F*_(1, 41)_ = 0.43, *p* = 0.65; dACC: *F*_(1, 40)_ = 0.30, *p* = 0.59]. There was no association between changes in hippocampal Gln, Glu, GSH and NAA and VO_2_max change [*F*_(1, 26)_ = 0.38, *p* = 0.55; *F*_(1, 26)_ = 0.42, *p* = 0.74; *F*_(1, 25)_ = 0.77, *p* = 0.52; *F*_(1, 25)_ = 0.54, *p* = 0.72]. In the dACC, Glu, Gln, and GSH changes [*F*_(1, 26)_ = 0.46, *p* = 0.64; *F*_(1, 26)_ = 0.30, *p* = 0.83; *F*_(1, 26)_ = 0.96, *p* = 0.43] were not associated with VO_2_max change, but increased VO_2_max was associated with increased dACC NAA levels [*F*_(1, 26)_ = 7.14, *p* = 0.01] (Supplementary Figure 2). While not significant, GM CBF showed a trend toward an association with VO_2_max change [*F*_(1, 45)_ = 3.06, *p* = 0.06]. No other associations with VO_2_max changes were found (Supplementary Table 3).

#### Peripheral Neurotrophic Factors

BDNF and IGF level changes were found to be positively associated with change in VO_2_max [*F*_(1, 35)_ = 6.84, *p* = 0.01; *F*_(1, 35)_ = 6.27, *p* < 0.01] (Supplementary Figure 2), whereas VEGF level changes were not [*F*_(1, 36)_ = 0.22, *p* = 0.63, *F*_(1, 36)_ = 1.09, *p* = 0.30].

## Discussion

We investigated the effects of a 12-week high- vs. low-intensity exercise intervention paradigm on various structural and functional brain changes. Despite adherence to the intervention in both groups (Figure 3C), we found that cardiovascular fitness increased significantly independent of the exercise intensity. Nevertheless, *post-hoc* tests revealed that this effect was driven by significant increases in the high-intensity group. While we did not find differential effects of exercise intensity on changes in hippocampal volume, vasculature, or metabolite measures, we found a significant decrease in the right hippocampal volume, an increase in left hippocampus GSH levels, and a decrease in left hippocampal CBV across conditions over time. However, these specific changes were not associated with individual changes in cardiorespiratory fitness. Instead, BDNF and IGF, as well as dACC NAA levels (as a control region), were positively associated with cardiorespiratory fitness changes.

### Hippocampal Volume and Its Relation to Exercise

We found decreased right hippocampal volume, particularly in the high-intensity exercise condition. This effect was not found to be driven by changes in specific hippocampal subfields as previously suggested (35). Although most studies, particularly in older adults, have reported increases in hippocampal volume following exercise (9, 12, 73), Wagner et al. (26) also demonstrated that young, healthy participants (age 21–28) who failed to benefit from an exercise program showed decreases in hippocampal volume. These findings suggest that changes in hippocampal volume are highly variable between individuals, especially in young adults (74). Indeed, a meta-analysis concluded that exercise does not stimulate the hippocampal growth in young participants but instead prevents its volume decline as it occurs with increasing age (11). Additionally, many studies that found hippocampal volume increases through exercise interventions in younger adults were conducted in patient populations instead of healthy individuals (75–79). Hence, exercise effects on hippocampal volume may be dependent on age and disease (80), and therefore exercise could be regarded as a restorative, rather than stimulatory intervention.

### Neuro-Metabolites, BDNF, and IGF and Their Relation to Exercise

To further understand the potential underpinnings of the volume reductions of the right hippocampus, we investigated neuro-metabolite concentrations and peripheral BDNF and IGF concentrations as markers of neuronal remodeling. Utilizing the potential of ultra-high field MRI, we resolved numerous (low-concentration) neuro-metabolites, such as Glu, Gln, and GSH additionally to NAA. This was important as previous studies in young adults reported both increases (81) and decreases (26) in Glx (Glu + Gln) and NAA after aerobic exercise.

We found increases in GSH levels in the left hippocampus over time, independent of exercise intensity, which were mainly driven by an increase in the low-intensity condition. GSH is known to be responsible for the survival and function of neural cells and for sustaining dendrite integrity and cognitive function (82). Reducing GSH levels in hippocampal neurons of mice resulted, e.g., in dendritic disruption, glial activation in CA1, and cognitive impairment (83). GSH levels have previously been shown to increase in rats after long-term exercise (84), but not in the hippocampus; therefore, this finding was somewhat surprising, particularly given the absence of findings in other metabolites involved in neuronal integrity in the hippocampus. We can speculate that increases in GSH levels detected here could be indicative of cell proliferation, but replication of these findings is needed, and associations with fitness changes will need to be confirmed. Furthermore, neuro-metabolite levels in this study were only measured in the left hippocampus, whereas volume decreases were found particularly in the right hippocampus. Previous studies have found metabolite changes due to exercise interventions in both the left (81) and right hippocampus (26), and therefore future studies should consider acquiring bilateral hippocampal MRS data.

Although no associations between fitness and hippocampal NAA were found, increased fitness was associated with increased NAA levels in the dACC (the control region). NAA is a well-accepted marker of neuronal viability, and exercise-induced increases in NAA could potentially reflect improvements in neuronal health. In the rodent hippocampus, NAA changes have been linked to neurogenesis (85–88), a form of structural hippocampal plasticity that was recently re-confirmed to take place also in the human brain (89–92) and is thought to underlie associations between exercise and hippocampal volume (9, 13, 24, 35, 61, 93). In our exploratory analyses, we found a novel positive association between NAA in the dACC and fitness. A previous study (81) has found changes in NAA in the hippocampus that we could not replicate. This exploratory finding thus requires further replication, in order to investigate whether exercise-induced alterations in neurometabolites are region-specific or global.

We further found individual BDNF and IGF changes to be associated with individual fitness changes. While BDNF has been suggested as a primary candidate, IGF also plays a vital role in stimulating neurogenesis in the hippocampus (63, 94). Most rodent studies determined hippocampal levels of BDNF and IGF, whereas we determined neurotrophic factors in peripheral blood, which may not directly reflect changes in the hippocampus or its subregions. Nevertheless, BDNF and IGF associations with fitness indicate that individual changes in cardiorespiratory fitness were indeed associated with markers of neuronal health.

In summary, we found exercise-independent increases of GSH, possibly indicating a gain in cell proliferation over time. Additionally, dACC NAA levels, as well as BDNF and IGF levels, were positively associated with fitness, hinting toward a relationship of cardiovascular fitness and neuronal remodeling in young, healthy adults.

### Changes in Vasculature and VEGF and Its Relation to Exercise

Additionally, vascularization was investigated as a potential underlying mechanism of exercise-induced changes in hippocampal volume. Changes in vascularization can be estimated using CBF, CBV, as well as the peripheral neurotrophic factor VEGF.

One of the first studies on exercise-induced changes in vascularization suggested strong, positive effects of exercise on hippocampal angiogenesis, estimated with CBV, in both rodents and older adults (16). They argued that oxygen and neurotrophic factors can reach the brain more efficiently through formations of new blood vessels and could, therefore, positively affect cardio-pulmonary and cognitive functions. Subsequent studies have not yet replicated beneficial exercise-induced changes in blood volume but found a positive association of fitness changes and changes in hippocampal vasculature (CBF and CBV) in a population of older adults (95). In contrast, Thomas et al. found no effects of exercise on CBV (12) and we found a decrease in left hippocampal CBV over time in young adults which is suggestive of no or negative effects of exercise on hippocampal vasculature.

Furthermore, even though non-significantly, increases in individual GM CBF were associated with improved individual fitness, indicating global, whole-brain changes rather than specific fitness effects on hippocampal CBF, but these results need replication. CBF is a potential marker for neuronal activity, as blood supply needs to be guaranteed during higher energy demands (96). These findings are in line with several other studies that found widespread beneficial effects of exercise on CBF (97, 98), and no effects on CBF in the hippocampus (99).

In line with the absence of exercise effects on hippocampal vasculature, we also did not find any exercise-induced changes in peripheral VEGF levels. VEGF is thought to play a pivotal role in the formation of new blood vessels. Its peripheral levels were not changed in our study, even though we used collection in PECT tubes, which should provide an accurate estimation of *in-vivo* circulating VEGF levels (100).

In sum, independent of fitness, we found CBV in the left hippocampus to decrease over time, as a potential marker for angiogenesis. Interestingly, even though non-significant, we found indications that increased fitness is positively associated with global increases in CBF.

### Important Factors in Exercise Research

Contrary to our hypothesis and previous studies, mainly performed in middle-aged or older adults (9, 101), our high-intensity aerobic exercise intervention did not improve cardiorespiratory fitness significantly more than the low-intensity stretching and toning intervention. Although related exceptions have been reported (102), this was an unexpected finding, as low-intensity training was previously found not to influence VO_2_max (101), and a meta-analysis stressed the importance of high-intensity training (HR > 80% of the maximum HR) for improving fitness in younger adults (18–45 years old) (33). Moreover, the two groups strictly adhered to the instructed intensities of the respective interventions, as is further evidenced by the significantly higher percentage of maximum HR attained during the training sessions in the high-intensity condition (Figure 3C). These findings, therefore, may suggest that engaging in stretching and toning activities might have caused sufficiently large changes in activity and/or lifestyle to increase fitness in young, otherwise non-athletic volunteers. Indeed, previous studies have argued that also low-intensity exercise such as yoga and pilates might be beneficial for cardiovascular fitness in young individuals (103, 104), even though sample sizes were small and effect sizes were medium. This would partially explain why we did not observe an interaction effect between the low- and high-intensity conditions (despite the fact that only the high-intensity group showed a significant increase in VO_2_max). Nevertheless, our findings suggest that perhaps in some individuals, even minimal time of elevated HR might be enough to increase cardiorespiratory fitness, which is an important finding for those starting to exercise again, or in rehabilitation medicine. However, this suggestion needs to be interpreted with caution and requires further investigation, as time spent in these high HR regimes did not explain changes in VO_2_max. As an alternative explanation, it has been suggested that some forms of exercise (regardless of their intensity) are more “mindful” than others, presuming possible synergistic effects of physical and cognitive activity on brain structure and function; however, this remains to be confirmed in future studies (105, 106).

On the other hand, however, other studies have found that moderately intense exercise benefits neuronal health most (107–109). Therefore, speculatively, our high-intensity intervention may have been too intense, which might have evoked an accompanying stress reaction, which in turn could have deteriorated, rather than improved, some of the measures studied here (110–112). Although we did not measure stress hormone levels, we found no association between cardiorespiratory fitness or volume measures and subjective stress scores (measured with the Depression Anxiety Stress Scale - DASS-S), indicating that this explanation is less likely.

Taken together, our findings highlight the importance of a passive control condition that refrains from exercise entirely in future studies; in addition to an active control condition (as implemented in this study), and different levels of exercise intensities (e.g., to control for possible environmental “enrichment” effects, including changes in social interactions, and individual motivation), to investigate if lifestyle changes or exercise training change brain health in young, healthy adults. In our study, we did not directly supervise the exercise sessions, but chose a more naturalistic approach, in which participants could choose from a predefined list of classes at the sports center. These classes were supervised by a certified fitness instructor. This approach has the advantage that it resembles better how exercise would be implemented in everyday life and introduces less stress and pressure. Nevertheless, it also meant that we had less control over the classes that participants chose to follow, which might explain some of the variance in the cardiovascular fitness measures in this study.

Notably, previous studies have been inconsistent in their operationalization of (maximal) cardiorespiratory fitness (e.g., VO_2_plateau, VO_2_peak, VO_2_vat), thereby making it challenging to compare results (12, 26, 29, 30, 39, 81, 93, 101, 113). Therefore, we warrant it essential to harmonize the analyses and detailed reportings of such outcome measures.

It has further been suggested that individuals differ in the extent to which they are susceptible to fitness-based interventions, which may subsequently also influence the relationship between exercise and brain-related changes (26, 93, 114, 115). Upon confirmation in more extensive studies [e.g., the IGNITE study (116)], it would become essential to develop individualized exercise programs to confer neurobiological benefits (11). So far, the most prominent advantages of exercise for the brain seem to apply mainly to middle-aged or older adults and diseased populations, and less so to younger, healthy adults, due to possible ceiling effects and an already optimal neuronal health. Nevertheless, it is important to mention that we studied a relatively homogenous group (comparable BMI, age, fitness), which might have made ceiling effects even more likely. Overall, our results point toward the hypothesis that exercise benefits on the human brain are restorative rather than stimulatory.

Interestingly, evidence is accumulating that exercise effects might be transient and change relatively rapidly in young adults. For instance, Van Der Borght et al. (117) found that the vasculature in rodents changed rapidly 3 days after exercise training but also declined again after 24 h of inactive behavior. Thomas et al. (12) even observed temporary changes in young healthy adults, at least for the anterior hippocampus. Specifically, they found a temporary effect of exercise intensity on the volume and myelination of the anterior hippocampus. As these changes were temporary, consistency and regularity of training seem to be essential factors, which could have influenced our measures. In our study, we intentionally controlled for rapid exercise effects on perfusion by guaranteeing a 24 h gap between the last exercise session and MRI measurements, as we were interested in the prolonged effects of exercise intensity.

## Conclusion

In sum, we found that cardiorespiratory fitness improved independent of exercise intensity in these young, non-athletic volunteers, but observed no differential effects of exercise intensity over time for hippocampal volume, vasculature, or neuro-metabolite measures. We found a fitness-independent decrease in the right hippocampal volume, an increase in GSH, and a decrease in CBV in the left hippocampus over time. In exploratory analyses, changes in BDNF and IGF levels, as well as dACC NAA levels, were found to be associated with individual cardiorespiratory fitness changes, indicating a beneficial effect of exercise on neuronal health on an individual level, independent of the exercise intervention intensity. All in all, the benefits of physical activity are likely not attributable to a single mechanism but probably involve multiple biological changes within the body and brain that could differ across individuals. In our study in a young population, exploratory analyses suggest that cardiovascular fitness shows positive associations with CBF and markers of neuronal viability, arguing that exercise does not seem to benefit the hippocampus specifically. Our findings highlight the utility of a multimodal approach in assessing exercise-induced neural integrity. Work of this kind will help to bridge the gap between animal and human studies by studying neuronal changes that occur on the macroscopic and microscopic level, as well as understand the role of exercise intensities to use physical activity as a potential future treatment for various disorders in humans.

## Data Availability Statement

The original contributions presented in the study are included in the article/Supplementary Material, further inquiries can be directed to the corresponding author/s.

## Ethics Statement

The studies involving human participants were reviewed and approved by the Medical-Ethical Committee of the Amsterdam University Medical Centre, University of Amsterdam. The patients/participants provided their written informed consent to participate in this study.

## Author Contributions

All authors made a substantial contribution to the concept and design, acquisition of data or analysis and interpretation of data, drafted the article or revised it critically for important intellectual content, and approved the version to be published.

## Funding

This study was funded by a project grant from Amsterdam Brain and Cognition (ABC) to LR and PL. PL and AS were supported by ABC and the Center for Urban Mental Health of the UvA, PL by Alzheimer's Nederland, AK and LR by Eurostars (estar19210), and AS by an NWO Veni grant (016.196.153).

## Conflict of Interest

The authors declare that the research was conducted in the absence of any commercial or financial relationships that could be construed as a potential conflict of interest.

## Publisher's Note

All claims expressed in this article are solely those of the authors and do not necessarily represent those of their affiliated organizations, or those of the publisher, the editors and the reviewers. Any product that may be evaluated in this article, or claim that may be made by its manufacturer, is not guaranteed or endorsed by the publisher.
