# Supplementary material for: A Randomized Controlled Trial on the Effects of a 12-Week High- vs. Low-Intensity Exercise Intervention on Hippocampal Structure and Function in Healthy, Young Adults
Source: Front Psychiatry. 2022 Jan 21;12:780095. doi: 10.3389/fpsyt.2021.780095 (PMC8814653; doi:10.3389/fpsyt.2021.780095)
Supplement: Supplementary file 1 [file Data_Sheet_1.PDF]

CERT ✓ Consensus on Exercise Reporting Template

A Checklist for what to include when reporting exercise programs

Section/Topic	Item #	Checklist item	Location ** Primary paper (page, table, appendix)	† Other (paper or protocol, website (URL)
**WHAT: materials**	1	Detailed description of the type of exercise equipment (e.g. weights, exercise equipment such as machines, treadmill, bicycle ergometer etc)	Suppl. Mat. 1.5	*1)
**WHO: provider**	2	Detailed description of the qualifications, teaching/supervising expertise, and/or training undertaken by the exercise instructor	Main text p.9	*2)
**HOW: delivery**	3	Describe whether exercises are performed individually or in a group	Main text p.9	*2)
	4	Describe whether exercises are supervised or unsupervised and how they are delivered	Main text p.9	*2)
	5	Detailed description of how adherence to exercise is measured and reported	Main text p.9	*3)
	6	Detailed description of motivation strategies	Main text p.9/10	*4)
	7a	Detailed description of the decision rule(s) for determining exercise progression	Suppl. Mat. 1.5	*3)
	7b	Detailed description of how the exercise program was progressed	Suppl. Mat. 1.5	*3)
	8	Detailed description of each exercise to enable replication (e.g. photographs, illustrations , video etc)	Suppl. Mat. 1.5	*1)
	9	Detailed description of any home program component (e.g. other exercises, stretching etc)	Suppl. Mat. 1.5	*5)
	10	Describe whether there are any non-exercise components (e.g. education, cognitive behavioural therapy, massage etc)	Suppl. Mat. 1.5	*5)
	11	Describe the type and number of adverse events that occurred during exercise	Suppl. Mat. 1.5	*6)

**WHERE: location**	12	Describe the setting in which the exercises are performed	Main text p.9	*1) & *7)
**WHEN, HOW MUCH: dosage**	13	Detailed description of the exercise intervention including, but not limited to, number of exercise repetitions/sets/sessions, session duration, intervention/program duration etc	Main text p.9 & Suppl. Met. 1.5	*1) & *7)
**TAILORING: what, how**	14a	Describe whether the exercises are generic (one size fits all) or tailored whether tailored to the individual	Main text p.9	*1) & *2)
	14b	Detailed description of how exercises are tailored to the individual	NA	
	15	Describe the decision rule for determining the starting level at which people commence an exercise program (such as beginner, intermediate, advanced etc)	NA	
**HOW WELL: planned, actual**	16a	Describe how adherence or fidelity to the exercise intervention is assessed/measured	Main text p.9 & Suppl. Met. 1.5	*3) & *4)
	16b	Describe the extent to which the intervention was delivered as planned	Main text p.17,18	*7)

***It is recommended that this checklist is used in conjunction with the Explanation and Elaboration Statement which is a guide each item in the CERT Checklist**

The CERT Checklist is designed for reporting details of an exercise intervention. The CERT Checklist should be used in conjunction with a reporting checklist appropriate for the study type e.g. the CONSORT Statement (www.consort-statement.org) for randomised controlled trials, the SPIRIT Statement (www.spirit-statement.org) for a clinical trial protocol. For further guidance regarding reporting guidelines please consult the EQUATOR network (www.equator-network.org)

** Authors – please use N/A if an item is not applicable   Reviewers – please use "?" if information is not provided or not/insufficiently reported

† If the information is not provided in the primary paper that is under consideration, please provide details of where this information is available e.g. in a published protocol, published papers (provide citation details) or on a website (provide the URL).

***1)** Recommended exercise group classes per exercise intensity group (translated from Dutch):

Low-Intensity Group:

• BBB - The class consists of a short warm-up followed by various exercises that focus on the belly, buttocks, and legs.

• Body power* - This is a group workout to music using simple and effective exercises with barbells and dumbbells. It is focused on the muscular endurance of the whole body.
• Essentrics - At Essentrics you get an effective toning workout to music using dynamic stretches and fluid movements without using gear. The main goals are a slimmer silhouette, more flexibility, and better posture.
• Pilates - In this class the focus is on: posture and control, flexibility, breathing, and awareness. In Pilates, you do floor exercises that target all postural muscles in the body, especially the abdominal and back muscles. You do the exercises slowly, fluently and in collaboration with your breath. You concentrate on doing the exercises carefully and accurately, and not on the number of repetitions. The result is better posture and flexibility.
• TrippleShape barre workout - Ballet Barre workout is a combination of ballet, pilates, and yoga to contemporary music.
• Yoga basic - This is a basic yoga class. The focus lies on: stretching and holding postures.
• Kinesis - Kinesis Training takes traditional exercises (like chest press, lat pull, row) and combines them with functional movements (like reaching, squatting, bending). It develops balance, core/overall strength, and flexibility for people of all fitness levels.
• Abs - 15 minutes of abs training using own-body weight.
• Basic movement - In this class good technique of the most basic exercises using own-body weight is being practiced. This lesson focuses on questions such as: How do I perform the squat correctly? What is a pull-up and how do I do it? What variations are there for a deadlift?
• Mobility class - This lesson focuses on increasing flexibility in muscles and joints.
• Calisthenics - This is a class to practice exercises like a handstand, muscle up, human flag, front and back lever.
• Core*- This lesson focuses on posture and strengthening the abdominal and back muscles in a controlled manner. The training does not only consist of floor exercises but is also offered in challenging circuit forms.
• W.A.C.* - Weightlifting Aerobic Circuit: this workout is originally based on Olympic weightlifting, a valuable full-body workout.

**For Core, W.A.C., and body power, participants were only allowed to do a maximum of 1 of these 3 per week for this sports program.*

High-Intensity Group:
• Combat - In this class you train your entire body during circuit training. It includes techniques from (kick) boxing, self-defense techniques, strength, and conditioning to increase your heart rate.
• Fitness training - This is circuit training in which all available materials are used. Walking and jumping are used a lot in this training, the intensity is largely determined by the participants themselves. A high-intensity training involving both strength and condition.
• Fit Fight - This is a powerful cardio workout inspired by Eastern martial arts such as karate, boxing, taekwondo, and Muay Thai to music.

• Kick & shape - This is an intensive class in which participants learn various punching and kicking techniques against the punching bag to music.
• Spinning - In this class participants sit on a spinning bike, on which, under the guidance of the teacher, they go on a 'bike tour' in which different speeds alternate. The resistance is chosen by the participants themselves.
• Step & dance - Step & dance is a choreographically challenging, advanced step class, with the goal of increasing aerobic fitness.
• Total body workout - This class is a short piece of aerobics hi/low impact after a warm-up. This is followed by exercises for all muscle groups: back, arms, abdomen, shoulders, chest, and legs. This class improves the participant's overall fitness, both cardio and muscle endurance are trained.
• Synrgy - Synrgy is a large device, with rods, ropes, bells, and whistles. Participants can follow group training sessions of 30 minutes several times a day, in which they do high-intensity interval training in circuit form.
• H.I.I.T. - High-Intensity Interval Training consists of short periods of intense effort, followed by short recovery moments. Participants train their general condition but also strength and speed, using simple materials or body weight.
• Crossfit - This small group training is based on the principles of a fitness concept from the USA: functionality, variation, and high intensity. Participants train speed, (muscle) endurance, strength, flexibility, and coordination.

***2)** In collaboration with the sports scientists of the University Sports Center (USC), we provided a list of generic exercise group classes offered at the USC which were supervised by an experienced fitness instructor, that participants were allowed to choose from (Supplementary Materials 1.5; *1)).

***3)** Their presence and active engagement were monitored by tracking their sports center visits using an automated fingerprint entrance system and by using weekly questionnaires on exercise duration and activities. Additionally, participants received a HR monitor (Polar, Finland) to measure HR during each training session (Table 1).

***4)** For motivation purposes, participants were contacted regularly to check in on their progress and one experimenter joined them at least once during the intervention period to train with them (more detail in Supplementary Material 1.5).

***5)** No home-program or non-exercise components were added to the intervention.

***6)** No adverse events occurred during the exercise intervention in this study.

***7)** All participants were enrolled in an exercise program for 12 weeks, in which they were instructed to exercise three times a week for 45 minutes (Haskell et al., 2007; Astorino et al., 2017) at the university sports center (USC).

***8)** Hours spent exercising demonstrated high compliance with the exercise program in both exercise groups (Table 1). As expected, participants in the high-intensity condition spent significantly more time exercising in the intended higher HR regime than the low-intensity condition, which did not explain the change in VO2max ($t(40)=1.34$, $p=0.19$; Figure 3C). The low- and high-intensity exercise groups did not show a significant change from pre to post-intervention on the total score or scores for walking and intermediate-intensity activities as measured with the IPAQ questionnaire. However, a significant condition x time effect was found on vigorous-intensity activities ($\chi^2(1)=5.46, p=0.02$), indicating an increase in the high-intensity group but not in the low-intensity group (Supplementary Results 2.3). Nevertheless, contrary to our expectations, we found no condition x time effect on VO2max; instead, we found decisive evidence (BF>100) for an effect of time ($\chi^2(1)=15.43, p<0.001$; low-intensity: 4.7%, high-intensity: 12.65% change) (Figure 3A). Nevertheless, post-hoc tests revealed only a significant increase in the high-intensity condition (low: $t(49)=1.72$, $p=0.09$; high: $t(49)=4.20$, $p<0.01$). In line with the results on VO2max, we found no interaction effect, but decisive evidence (BF>100) for a main effect of time ($\chi^2(1)=38.92, p<0.001$) on the maximal resistance attained. Post-hoc tests revealed a significant increase in both conditions ($t(23)=4.67, p<0.01$; $t(24)=7.02, p<0.01$; Figure 3B). No effects on HR during the VO2max test were found (Supplementary Table 2).
