# Supplementary material for: A Randomized Controlled Trial on the Effects of a 12-Week High- vs. Low-Intensity Exercise Intervention on Hippocampal Structure and Function in Healthy, Young Adults
Source: Front Psychiatry. 2022 Jan 21;12:780095. doi: 10.3389/fpsyt.2021.780095 (PMC8814653; doi:10.3389/fpsyt.2021.780095)
Supplement: Supplementary file 2 [file Data_Sheet_2.pdf]

Supplements

1. Supplementary Methods

1.1. 1H-MRS:

Supplementary Figure 1 | Representative spectra for one participant, measured in the **A)** hippocampus and **B)** dorsal anterior cingulate cortex (dACC).

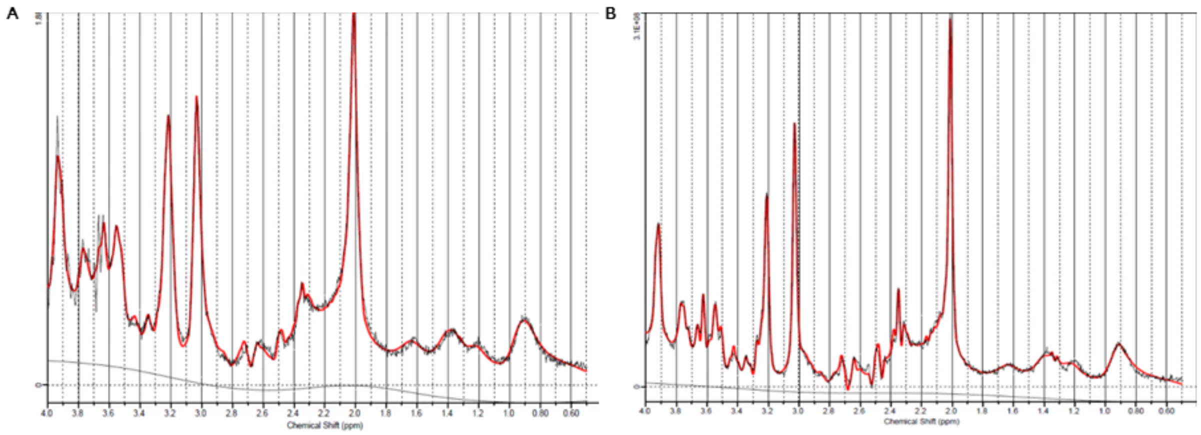

Spectra were fitted using LCModel with a simulated basis set with a measured macromolecular baseline (using an sLASER dual inversion recovery sequence(Penner and Bartha, 2015)) and the following metabolites: alanine (Ala), aspartate (Asp), creatine (Cr), γ-aminobutyric acid (GABA), Gln, Glu, GSH, glycine (Gly), glycerophosphocholine (GPC), Inositol, Lac, phosphoethanolamine (PE), phosphocholine (PCh), phosphocreatine (PCr), inositol (Ins), choline (Cho), NAA, N-acetyl aspartyl glutamate (NAAG), taurine (Tau), scylloinositol (Sci), succinate (Suc), pyruvate (Pyr) and threonine (Thr).

The following control parameters were used for LCModel analyses:

```
deltat= 2.50e-04
 doecc= F
 dows= T
 hzpppm= 2.9804e+02
 nunfil= 2048
 ppmend= 0.5
 ppmst= 4.0
 DKNTMN= 0.5
 atth2o= 0.7
 attmet= 1.0
 chcomb(17)= 'Lac+Thr'
 chcomb(18)= 'Cho+GPC+PC'
 chomit(1)= 'MM09'
 chomit(2)= 'MM12'
 chomit(3)= 'MM14'
 chomit(4)= 'MM17'
 chomit(5)= 'MM20'
```

```
chomit(6)= 'Ala'
chomit(7)= 'Pyr'
chomit(8)= 'Suc'
ncombi= 18
neach= 50
nomit= 8
wconc= individual values
CHUSE1(1)='NAA'
CHUSE1(2)='PCr'
CHUSE1(3)='PC'
```

1.2. Statistical Analysis

Supplementary Table 1 | Evidence Categories for Bayes Factor BF (Jeffrey, 1961; Wagenmakers, 2007)

Bayes Factor	Interpretation
> 100	Decisive evidence for H_A
30 - 100	Very strong evidence for H_A
10 - 30	Strong evidence for H_A
3 - 10	Substantial evidence for H_A
1 - 3	Anecdotal evidence for H_A
1	No evidence
1/3 - 1	Anecdotal evidence for H_0
1/10 – 1/3	Substantial evidence for H_0
1/30 – 1/10	Strong evidence for H_0
1/100 – 1/30	Very strong evidence for H_0
< 1/100	Decisive evidence for H_0

1.3. Sample Size Calculations

We aimed to investigate the neural mechanisms underlying brain volume changes resulting from exercise interventions. First, we determined the sample size to assess a hypothesized effect on neurometabolite signaling. A previous study investigating exercise effects on anterior cingulate cortex glutamate concentration changes reported an effect size of d=1.48 (Cohen d) in young participants (Maddock et al., 2016). As this study used an acute exercise intervention, we expected a more moderate effect size for a longitudinal exercise intervention of d=1.0. To detect the effect of exercise, with a power of 80% and an α of 0.05, we would need 12 participants per

exercise intervention group. Taking a 20% dropout into account (due to MRI data quality), we would need at least 15 subjects in each exercise intervention group.
Secondly, we calculated the required sample size to assess the effect of exercise on vascular outcome measures. A previous study using contrast-enhanced MRI assessed the effect of a dietary intervention with flavanol on CBV in the hippocampus (Brickman et al., 2014). This study detected an increase in CBV with an effect size of d=0.75. We expect the dietary intervention to have effects of a similar magnitude as the exercise intervention. With an effect size of d=0.75, a power of 80%, and an α of 0.05, we would need 16 subjects to detect a difference before and after the exercise intervention. Taking a 20% dropout into account, we would need at least 20 subjects in each exercise group.

1.4. CONSORT flow chart

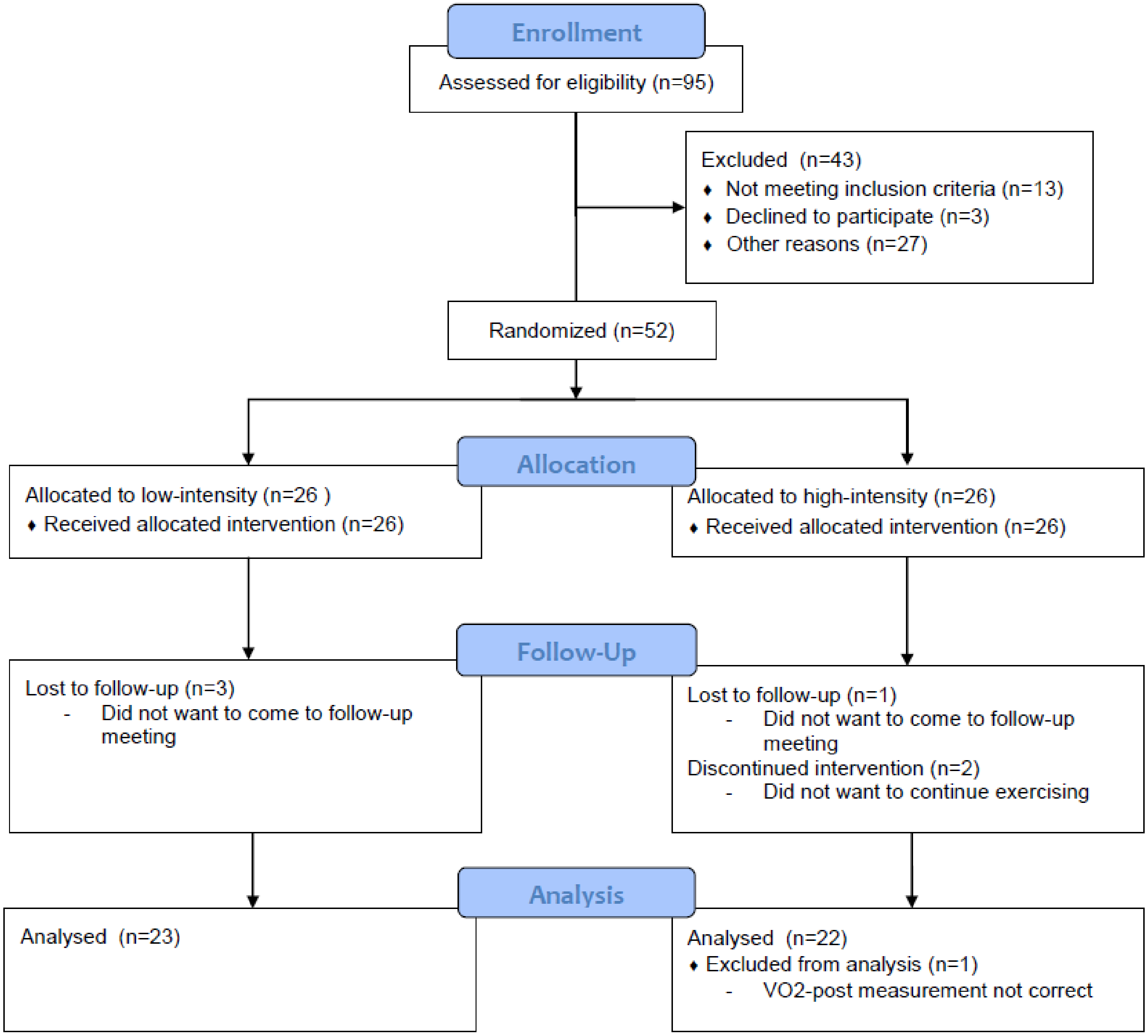

1.5. Exercise intervention

Recommended exercise group classes per exercise intensity group (translated from Dutch):

Low-Intensity Group:

• BBB - The class consists of a short warm-up followed by various exercises that focus on the belly, buttocks, and legs.

• Body power* - This is a group workout to music using simple and effective exercises with barbells and dumbbells. It is focused on the muscular endurance of the whole body.

• Essentrics - At Essentrics you get an effective toning workout to music using dynamic stretches and fluid movements without using gear. The main goals are a slimmer silhouette, more flexibility, and better posture.

• Pilates - In this class the focus is on: posture and control, flexibility, breathing, and awareness. In Pilates, you do floor exercises that target all postural muscles in the body, especially the abdominal and back muscles. You do the exercises slowly, fluently and in collaboration with your breath. You concentrate on doing the exercises carefully and accurately, and not on the number of repetitions. The result is better posture and flexibility.

• TrippleShape barre workout - Ballet Barre workout is a combination of ballet, pilates, and yoga to contemporary music.

• Yoga basic - This is a basic yoga class. The focus lies on: stretching and holding postures.

• Kinesis - Kinesis Training takes traditional exercises (like chest press, lat pull, row) and combines them with functional movements (like reaching, squatting, bending). It develops balance, core/overall strength, and flexibility for people of all fitness levels.

• Abs - 15 minutes of abs training using own-body weight.

• Basic movement - In this class good technique of the most basic exercises using own-body weight is being practiced. This lesson focuses on questions such as: How do I perform the squat correctly? What is a pull-up and how do I do it? What variations are there for a deadlift?

• Mobility class - This lesson focuses on increasing flexibility in muscles and joints.

• Calisthenics - This is a class to practice exercises like a handstand, muscle up, human flag, front and back lever.

• Core*- This lesson focuses on posture and strengthening the abdominal and back muscles in a controlled manner. The training does not only consist of floor exercises but is also offered in challenging circuit forms.

• W.A.C.* - Weightlifting Aerobic Circuit: this workout is originally based on Olympic weightlifting, a valuable full-body workout.

***For Core, W.A.C., and body power, participants were only allowed to do a maximum of 1 of these 3 per week for this sports program.**

High-Intensity Group:

• Combat - In this class you train your entire body during circuit training. It includes techniques from (kick) boxing, self-defense techniques, strength, and conditioning to increase your heart rate.

• Fitness training - This is circuit training in which all available materials are used. Walking and jumping are used a lot in this training, the intensity is largely determined by the participants themselves. A high-intensity training involving both strength and condition.

• Fit Fight - This is a powerful cardio workout inspired by Eastern martial arts such as karate, boxing, taekwondo, and Muay Thai to music.

• Kick & shape - This is an intensive class in which participants learn various punching and kicking techniques against the punching bag to music.

• Spinning - In this class participants sit on a spinning bike, on which, under the guidance of the teacher, they go on a 'bike tour' in which different speeds alternate. The resistance is chosen by the participants themselves.

• Step & dance - Step & dance is a choreographically challenging, advanced step class, with the goal of increasing aerobic fitness.

• Total body workout - This class is a short piece of aerobics hi/low impact after a warm-up. This is followed by exercises for all muscle groups: back, arms, abdomen, shoulders, chest, and legs. This class improves the participant’s overall fitness, both cardio and muscle endurance are trained.

• Synrgy - Synrgy is a large device, with rods, ropes, bells, and whistles. Participants can follow group training sessions of 30 minutes several times a day, in which they do high-intensity interval training in circuit form.

• H.I.I.T. - High-Intensity Interval Training consists of short periods of intense effort, followed by short recovery moments. Participants train their general condition but also strength and speed, using simple materials or body weight.

• Crossfit - This small group training is based on the principles of a fitness concept from the USA: functionality, variation, and high intensity. Participants train speed, (muscle) endurance, strength, flexibility, and coordination.

Details about the exercise intervention:

Exercise progression was monitored by tracking their sports center visits using an automated fingerprint entrance system and by using weekly questionnaires on exercise duration and activities. Additionally, participants received an HR monitor (Polar, Finland) to measure HR during each training session. In case the regularity of participants’ training sessions was

decreasing, participants were contacted and motivated to continue exercising by one of the experimenters.

No home-program or non-exercise components were added to the intervention. No adverse events occurred during the exercise intervention in this study.

2. Supplementary Results

2.1. Quality Control Spectra

Quality metric	Left Hippocampus<br>BL<br>*Mean ± std*	Post<br>*Mean ± std*	dACC<br>BL<br>*Mean ± std*	Post<br>*Mean ± std*
SNR	30.76 ± 6.05	28.85 ± 7.03	46.64 ± 2.96	47.41 ± 2.79
FWHM	14.41 ± 2.26	15.68 ± 3.71	7.55 ± 0.71	7.55 ± 0.72
Glu CRLB	3.17 ± 0.66	3.49 ± 0.76	2.0 ± 0	2.02 ± 0.15
Gln CRLB	29.41 ± 6.19	27.91 ± 6.69	19.97 ± 6.04	20.72 ± 5.98
GSH CRLB	7.48 ± 1.91	7.13 ± 1.49	7.67 ± 0.81	7.74 ± 0.83
NAA CRLB	1.02 ± 0.15	1.05 ± 0.22	1.0 ± 0	1.0 ± 0

2.2. Exploratory Analysis:

Supplementary Table 2 | Statistical tests for all variables: Linear mixed-effects models were used to investigate the condition (high- vs. low-intensity exercise) x time (pre- vs. post-intervention) x sex (female vs. male) interaction in Rv.3.5.3 using the lme4 package. Multiple comparison corrections using Sidak's resulted in an $\alpha^*=0.02$. Model selection was based on an adjusted top-down procedure, in which the resulting models were compared using the Bayesian information criterion (BIC); the model was consequently tested using chi-square (χ^2) tests.

Variables	*Interaction effects*	*Main effects*		*Tukey Post-Hoc test*
	Group * Time	***Time***	***Sex***	
Fitness measures				
VO2max	χ^2(5)=4.51, p=0.48, ΔBIC=-18.42	χ^2(1)=15.43, **p<0.001**, ΔBIC=11.15	χ^2(1)=21.26, **p<0.001**, ΔBIC=17.32	Female<Male; pre<post
Bike-resistance	χ^2(5)=11.84, p=0.04, ΔBIC=-11.09	χ^2(1)=38.92, **p<0.001**, ΔBIC=34.34	χ^2(1)=36.23, **p<0.001**, ΔBIC=31.64	Female<Male; pre<post
HR during VO2max	χ^2(7)=1.35, p=0.25, ΔBIC=-3.21	χ^2(1)=4.03, p=0.04, ΔBIC=-0.53	χ^2(1)<0.01, p=0.97, ΔBIC=-4.56	-
Volume measures				
Left Hippocampus				
Whole	χ^2(6)=5.70, p=0.45, ΔBIC=-21.54	χ^2(1)=2.68, p=0.10, ΔBIC=-1.82	χ^2(1)=24.97, **p<0.001**, ΔBIC=21.54	Female<Male
Dentate Gyrus	χ^2(6)=4.61, p=0.59, ΔBIC=-22.39	χ^2(1)=2.14, p=0.14, ΔBIC=-2.35	χ^2(1)=18.23, **p<0.001**, ΔBIC=14.27	Female<Male
CA1	χ^2(6)=2.68, p=0.85, ΔBIC=-24.20	χ^2(1)= 0.08, p=0.77, ΔBIC=-4.40	χ^2(1)= 23.46, **p<0.001**, ΔBIC=19.00	Female<Male
CA3	χ^2(6)=2.08, p=0.91, ΔBIC=-9.92	χ^2(1)= 0.95, p=0.33, ΔBIC=-1.05	χ^2(1)= 15.75, **p<0.001**, ΔBIC=11.26	Female<Male
WM	χ^2(5)=7.55, p=0.18, ΔBIC=-14.83	χ^2(1)= 5.18, **p=0.02**, ΔBIC=0.69	χ^2(1)= 6.31, **p=0.01**, ΔBIC=1.83	Male<Female; Post<pre
GM	χ^2(6)=2.83, p=0.83, ΔBIC=-24.17	χ^2(1)= 0.04, p=0.83, ΔBIC=-4.46	χ^2(1)= 31.79, **p<0.001**, ΔBIC=27.28	Female<Male
Right Hippocampus				
Whole	χ^2(5)=5.00, p=0.43, ΔBIC=-18.39	χ^2(1)=8.47, **p<0.01**, ΔBIC=4.03	χ^2(1)= 22.72, **p<0.001**, ΔBIC=18.22	Female<Male; Post<pre
Dentate Gyrus	χ^2(6)=10.59, p=0.10, ΔBIC=-16.41	χ^2(1)= 4.08, p=0.14, ΔBIC=-2.08	χ^2(1)= 13.04, **p<0.001**, ΔBIC=8.54	Female<Male
CA1	χ^2(6)=7.45, p=0.28, ΔBIC=-5.40	χ^2(1)= 0.84, p=0.36, ΔBIC=-4.30	χ^2(1)= 23.46, **p<0.001**, ΔBIC=20.60	Female<Male
CA3	χ^2(6)=2.08, p=0.91, ΔBIC=-24.85	χ^2(1)= 0.95, p=0.33, ΔBIC=-3.66	χ^2(1)= 15.75, **p<0.001**, ΔBIC=11.26	Female<Male
WM	χ^2(6)=10.22, p=0.12, ΔBIC=-6.72	χ^2(1)= 2.89, p=0.08, ΔBIC=-0.4	χ^2(1)= 5.34, **p=0.02**, ΔBIC=0.96	Male<Female
GM	χ^2(6)=3.74, p=0.71, ΔBIC=-23.26	χ^2(1)= 0.50, p=0.48, ΔBIC=-4.00	χ^2(1)= 27.06, **p<0.001**, ΔBIC=23.44	Female<Male
dACC				
	χ^2(6)=7.03, p=0.95, ΔBIC=-20.10	χ^2(1)= 0.005, p=0.95, ΔBIC=-5.5	χ^2(1)= 5.4, **p=0.02**, ΔBIC=1.1	Female<Male
1H-MRS				
Hippocampus				
GLU	χ^2(7)=4.22, p=0.74, ΔBIC=-26.44	χ^2(1)=0.82, p=0.36, ΔBIC=-3.55	χ^2(1)=0.84, p=0.36, ΔBIC=-3.53	-
GLN	χ^2(6)=4.60, p=0.60, ΔBIC=-21.54	χ^2(1)=1.92, p=0.17, ΔBIC=-3.56	χ^2(1)=4.57, *p=0.03*, ΔBIC=0.21	*Female<Male*
NAA	χ^2(7)=6.37, p=0.50, ΔBIC=-24.13	χ^2(1)=2.71, p=0.10, ΔBIC=-1.65	χ^2(1)=0.32, p=0.57, ΔBIC=-4.04	-
GSH	χ^2(6)=6.37, p=0.46, ΔBIC=-21.46	χ^2(1)=9.21, **p<0.01**, ΔBIC=4.83	χ^2(1)=2.72, p=0.09, ΔBIC=-2.65	Pre<post
dACC				
GLU	χ^2(6)=10.48, p=0.11, ΔBIC=-16.32	χ^2(1)=1.85, p=0.17, ΔBIC=-2.61	χ^2(1)=8.87, **p<0.01**, ΔBIC=4.41	Male<Female
GLN	χ^2(7)=7.59, p=0.37, ΔBIC=-23.67	χ^2(1)=0.01, p=0.91, ΔBIC=-5.05	χ^2(1)=2.43, p=0.12, ΔBIC=-2.03	-
NAA	χ^2(6)=5.52, p=0.48, ΔBIC=-22.72	χ^2(1)=3.77, p=0.05, ΔBIC=-1.3	χ^2(1)=6.05, **p=0.01**, ΔBIC=5.95	Male<Female
GSH	χ^2(7)=5.68, p=0.58, ΔBIC=-34.41	χ^2(1)=0.05, p=0.83, ΔBIC=-3.69	χ^2(1)=2.27, p=0.13, ΔBIC=-2.81	-
Vasculature				
CBF				
Left Hippocampus	χ^2(6)=5.59, p=0.47, ΔBIC=-21.13	χ^2(1)=0.19, p=0.66, ΔBIC=-4.33	χ^2(1)=0.74, p=0.39, ΔBIC=-4.32	-
Right Hippocampus	χ^2(7)=5.65, p=0.58, ΔBIC=-25.36	χ^2(1)=0.95, p=0.33, ΔBIC=-3.48	χ^2(1)<0.01, p=0.94, ΔBIC=-4.42	-
GM	χ^2(7)=13.60, p=0.06, ΔBIC=-18.67	χ^2(1)=2.84, p=0.09, ΔBIC=-2.42	χ^2(1)=0.85, p=0.36, ΔBIC=-4.43	-
CBV				
Left Hippocampus	χ^2(6)=4.34, p=0.63, ΔBIC=-22.24	χ^2(1)=5.97, **p=0.01** ΔBIC=2.05	χ^2(1)=15.21, **p<0.01**, ΔBIC=10.78	Male<Female
Right Hippocampus	χ^2(6)=6.86, p=0.33, ΔBIC=-20.21	χ^2(1)=3.57, p=0.06, ΔBIC=-1.12	χ^2(1)=6.45, **p=0.01**, ΔBIC=2.00	Male<Female
GM	χ^2(7)=8.23, p=0.31, ΔBIC=-22.70	χ^2(1)=0.98, p=0.32, ΔBIC=-3.43	χ^2(1)=1.71, p=0.19, ΔBIC=-3.31	-
Myelination (R1)				
WM left Hippocampus	χ^2(7)=8.90, p=0.26, ΔBIC=-23.1	χ^2(1)=0.20, p=0.65, ΔBIC=-5.6	χ^2(1)=0.32, p=0.57, ΔBIC=-5.7	-
WM right Hippocampus	χ^2(7)=12.76, p=0.08, ΔBIC=-21.0	χ^2(1)=0.42, p=0.52, ΔBIC=-4.1	χ^2(1)=0.63, p=0.43, ΔBIC=-4.1	-
Neurotrophic factors				
BDNF	χ^2(7)=11.06, p=0.14, ΔBIC=-20.20	χ^2(1)=0.95, p=0.33, ΔBIC=-4.50	χ^2(1)=0.05, p=0.82, ΔBIC=-4.40	-
VEGF	χ^2(7)=8.10, p=0.32, ΔBIC=-23.32	χ^2(1)=0.40, p=0.53, ΔBIC=-4.09	χ^2(1)=0.83, p=0.36, ΔBIC=-4.34	-
IGF	χ^2(7)=4.49, p=0.72, ΔBIC=-17.06	χ^2(1)=0.01, p=0.94, ΔBIC=-5.52	χ^2(1)=1.59, p=0.21, ΔBIC=-3.10	-

Supplementary Table 3 | Exploratory analyses testing the association of all variables with VO2max, and hippocampal or dACC volume change were conducted using linear models in R, including the baseline measure of the explanatory variables, VO2max, and sex as covariates (α=0.05).

Variables	**Regression with VO2max**
SPORT	
VO2max	-
Bike-resistance	F(1,38)=7.46, **p=0.01**, ß=1.68
Volume measures	
Left Hippocampus	
Whole	F(1,41)=0.51, p=0.48, ß=1.74
Dentate Gyrus	F(1,42)=1.81, p=0.19, ß=0.38
CA1	F(1,40)=0.82, p=0.37, ß=-0.95
CA3	F(1,41)=0.88, p=0.36, ß=0.17
WM	F(1,37)=0.03, p=0.86, ß=-0.43
GM	F(1,38)=0.10, p=0.75, ß=-1.06
Right Hippocampus	
Whole	F(1,40)=0.51, p=0.48, ß=2.18
Dentate Gyrus	F(1,42)=1.80, p=0.19, ß=1.76
CA1	F(1,40)=0.08, p=0.78, ß=0.33
CA3	F(1,41)=0.88, p=0.36, ß=-0.20
WM	F(1,37)=0.04, p=0.84, ß=0.46
GM	F(1,36)=1.22, p=0.28, ß=-3.47
dACC	F(1,40)=0.30, p=0.59, ß=3.22
1H-MRS	
Hippocampus	
GLU	F(1,26)<0.01,p=0.95, ß=-0.002
GLN	F(1,27)=0.38, p=0.55, ß=0.02
NAA	F(1,25)=0.07, p=0.80, ß=-0.006
GSH	F(1,25)=0.99, p=0.33, ß=0.007
dACC	
GLU	F(1,25)=0.08, p=0.78, ß=0.006
GLN	F(1,26)<0.01, p=0.98, ß=0.001
NAA	F(1,27)=7.14, **p=0.01, ß=0.04**
GSH	F(1,26)=0.26, p=0.61, ß=-0.002
Vasculature	
CBF	
Left Hippocampus	F(1,27)=1.08, p=0.31, ß=-0.002
Right Hippocampus	F(1,27)<0.01, p=0.96, ß=0.001
GM	F(1,28)=3.06, *p=0.06*, ß=0.35
CBV	
Left Hippocampus	F(1,27)=0.63, p=0.44, ß=-0.01
Right Hippocampus	F(1,27)=1.86, p=0.18, ß=-0.02
GM	F(1,27)<0.01, p=0.94, ß=-0.01
Myelination (R1)	
Left	F(1,37)=0.02, p=0.88, ß=0.001
Right	F(1,36)=0.33, p=0.57, ß=-0.001
Neurotrophic factors	
BDNF	F(1,36)=6.84, **p=0.01, ß=23.01**
VEGF	F(1,36)=0.02, p=0.89, ß=-0.04
IGF	F(-1,36)=5.19, **p=0.03, ß=0.02**

Supplementary Table 4 | Mean and standard deviation for pre- and post-exercise intervention per variable.

	HIGH-INTENSITY		*LOW-INTENSITY*	
	PRE	*POST*	*PRE*	*POST*
	Mean ± SD	*Mean ± SD*	*Mean ± SD*	*Mean ± SD*
Participant characteristics:				
Age (years)	23.12 ± 2.52	23.25 ± 2.31	24.19 ± 3.25	24.43 ± 3.14
Body Mass Index (kg/m²)	23.30 ± 2.80	22.96 ± 2.75	23.96 ± 3.24	23.78 ± 3.17
Fitness measures:				
VO2max (kg/mL/min.)	37.21 ± 7.39	41.60 ± 6.69	37.32 ± 6.58	39.39 ± 7.38
Max. Heart Rate (beats/min.)	186.60 ± 8.41	186.01 ± 8.26	189.12 ± 7.53	187.48 ± 7.31
Resistance pre (watts)	240.33 ± 61.94	273.54 ± 67.56	244.62 ± 45.54	262.17 ± 45.45
Volume measures:				
Left hippocampus	3025.33 ± 428.42	3024.76 ± 432.52	3082.54 ± 350.55	3076.88 ± 346.14
Right hippocampus	3128.94 ± 484.38	3123.99 ± 461.99	3095.15 ± 334.10	3063.12 ± 335.44
dACC	3984.78 ± 832.14	4053.26 ± 848.16	4241.57 ± 682.49	4254.65 ± 875.18
Vasculature				
CBF				
Left Hippocampus	42.24 ± 7.85	43.81 ± 5.42	47.03 ± 9.40	44.88 ± 7.75
Right Hippocampus	41.08 ± 7.89	42.73 ± 6.07	45.28 ± 9.15	42.90 ± 8.17
GM	46.41 ± 7.29	46.61 ± 6.41	48.44 ± 6.43	46.33 ± 7.02
CBV				
Left Hippocampus	2.19 ± 0.96	2.15 ± 0.85	2.11 ± 1.08	1.74 ± 0.59
Right Hippocampus	2.13 ± 0.78	1.93 ± 0.75	1.90 ± 0.77	1.82 ± 0.56
GM	2.44 ± 0.57	2.86 ± 0.84	2.49 ± 0.43	2.59 ± 0.64
Myelination (R1)				
Left	8.49E-04 ± 2.90E-05	8.49E-04 ± 2.90E-05	8.51E-04 ± 3.97E-05	8.41E-04 ± 2.28E-05
Right	8.47E-04 ± 3.75E-05	8.47E-04 ± 3.75E-05	8.60E-04 ± 4.85E-05	8.49E-04 ± 3.00E-05
1H-MRS				
Hippocampus				
GLU	7.80 ± 0.77	7.80 ± 0.65	7.60 ± 0.51	7.75 ± 0.54
GLN	1.17 ± 0.56	1.31 ± 0.67	1.06 ± 0.39	1.28 ± 0.59
NAA	1.79 ± 0.38	1.92 ± 0.30	1.71 ± 0.31	1.97 ± 0.64
GSH	10.23 ± 0.99	10.20 ± 0.48	9.95 ± 0.55	10.12 ± 0.31
dACC				
GLU	11.67 ± 0.91	11.75 ± 0.93	11.89 ± 0.66	11.98 ± 0.60
GLN	1.56 ± 0.51	1.56 ± 0.48	1.53 ± 0.34	1.46 ± 0.40
NAA	0.92 ± 0.07	0.92 ± 0.10	0.90 ± 0.31	0.91 ± 0.63
GSH	10.88 ± 0.76	10.93 ± 0.69	10.87 ± 0.64	11.10 ± 0.08

Supplementary Figure 2 | Exploratory analysis – associations with VO2max: Significant associations of the change in power output during the VO2max test, NAA concentration in the dACC, blood BDNF levels, and blood IGF levels with change in VO2max.

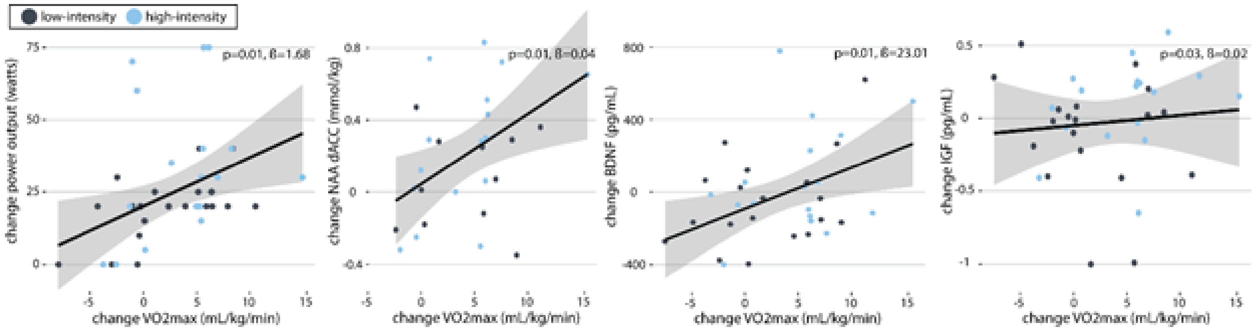

2.3. Physical activity besides the intervention:

Minutes spent on physical activity did not change for either low- or high-intensity condition from pre to post (low: t(37)=0.26, p=0.79; high: t(36)=-0.12, p=0.91). When splitting these hours up into different categories we also did not find any changes from pre to post intervention in: minutes spent walking (low: t(43)=0.85, p=0.40; high: t(45)=1.43, p=0.16); minutes spent on intermediate activities (low: t(40)=0.80, p=0.43; high: t(41)=0.77, p=0.45).

Minutes spent on vigorous activities significantly changed only in the high-intensity group (low: t(43)=-0.24, p=0.81; high: t(47)=3.46, p<0.01).

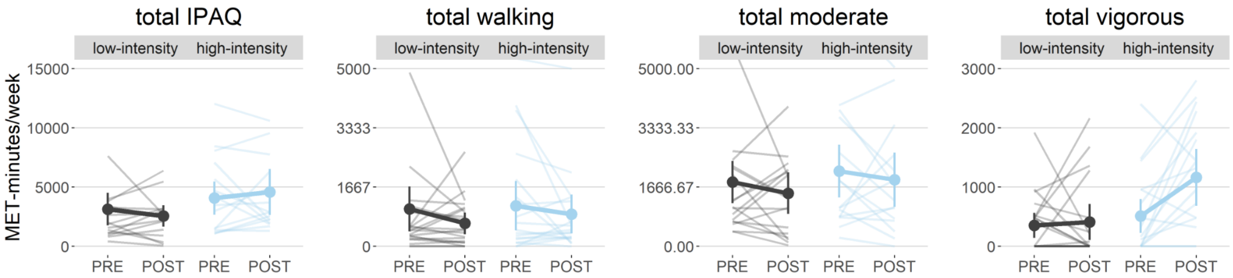

3. Supplementary References

Beck AT, Ward C, Mendelson M, Mock J, Erbaugh J. 1961. Beck depression inventory (BDI). Arch Gen Psychiatry 4:562–571.

de Beurs E, van Dyck R, Marquenie LA, Lange A, Blonk RWB. 2001. De DASS: een vragenlijst voor het meten van depressie, angst en stress. Gedragstherapie 1:35–54.

Brock Kirwan C, Hartshorn A, Stark SM, Goodrich-Hunsaker NJ, Hopkins RO, Stark CEL. 2012. Pattern separation deficits following damage to the hippocampus. Neuropsychologia 50:2408–2414.

Carver CS, White TL. 1994. Behavioral Inhibition, Behavioral Activation, and Affective Responses to Impending Reward and Punishment: The BIS/BAS Scales. J Pers Soc Psychol 67:319–333.

Eriksen BA, Eriksen CW. 1974. Effects of noise letters upon the identification of a target letter in a nonsearch task. Percept Psychophys [Internet] 16:143–149. Available from: https://link.springer.com/article/10.3758/BF03203267

Fujii T, Saito DN, Yanaka HT, Kosaka H, Okazawa H. 2014. Depressive mood modulates the anterior lateral CA1 and DG/CA3 during a pattern separation task in cognitively intact individuals: A functional MRI study. Hippocampus [Internet] 24:214–224. Available from: http://doi.wiley.com/10.1002/hipo.22216

Gaumer Erickson AS, Noonan PM. 2018. Self-efficacy formative questionnaire. In: The skills that matter: Teaching interpersonal and intrapersonal competencies in any classroom. . p 175–176.

Hartman DE. 2009. Test review Wechsler Adult Intelligence Scale IV (WAIS IV): Return of the gold standard. Appl Neuropsychol [Internet] 16:85–87. Available from: https://www.tandfonline.com/doi/abs/10.1080/09084280802644466

Jeffrey H. 1961. The Theory of Probability. Available from: https://books.google.nl/books?hl=nl&lr=&id=vh9Act9rtzQC&oi=fnd&pg=PA1&ots=feYALYY1nW&sig=18DCw5ZHgMYz8SO5WKZp_ttZRtI&redir_esc=y#v=onepage&q&f=false

Marteau TM, Bekker H. 1992. The development of a six-item short-form of the state scale of the Spielberger State-Trait Anxiety Inventory (STAI). Br J Clin Psychol [Internet] 31:301–306. Available from: http://doi.wiley.com/10.1111/j.2044-8260.1992.tb00997.x

Penner J, Bartha R. 2015. Semi-LASER 1H MR spectroscopy at 7 Tesla in human brain: Metabolite quantification incorporating subject-specific macromolecule removal. Magn Reson Med 74:4–12.

Saan RJ, Deelman BG. 1986. The 15-Words Test A and B.(a preliminary manual). Groningen Univ Groningen, Dep Neuropsychol.

Skinner EI, Fernandes MA. 2007. Neural correlates of recollection and familiarity: A review of neuroimaging and patient data. Neuropsychologia 45:2163–2179.

Snaith RP, Hamilton M, Morley S, Humayan A, Hargreaves D, Trigwell P. 1995. A scale for the assessment of hedonic tone. The Snaith-Hamilton Pleasure Scale. Br J Psychiatry 167:99–103.

de Sonneville LMJ. 1999. Amsterdam Neuropsychological Tasks: A computer-aided assessment program. In: Cognitive Ergonomics, Clinical Assessment and Computer-assisted Learning. . p 187–203.

Stark SM, Kirwan CB, Stark CEL. 2019. Mnemonic Similarity Task: A Tool for Assessing Hippocampal Integrity. Trends Cogn Sci 23:938–951.

Suwabe K, Hyodo K, Byun K, Ochi G, Yassa MA, Soya H. 2017. Acute moderate exercise improves mnemonic discrimination in young adults. Hippocampus [Internet] 27:229–234. Available from: http://doi.wiley.com/10.1002/hipo.22695

Wagenmakers EJ. 2007. A practical solution to the pervasive problems of p values. Psychon Bull Rev [Internet] 14:779–804. Available from: https://link.springer.com/article/10.3758/BF03194105

Yassa MA, Mattfeld AT, Stark SM, Stark CEL. 2011. Age-related memory deficits linked to circuit-specific disruptions in the hippocampus. Proc Natl Acad Sci U S A [Internet] 108:8873–8878. Available from: www.pnas.org/cgi/doi/10.1073/pnas.1101567108
